# Trade-off between accumulation potential and transmission efficiency in hypovirus variants infecting phytopathogenic fungi

**DOI:** 10.1128/mbio.02922-25

**Published:** 2026-01-21

**Authors:** Shian Yang, Ruoyin Dai, Shujing Liu, Tianxing Pang, Shujuan Gong, Mengyuan Tian, Zhensheng Kang, Hongying Chen, Ming Luo, Ida Bagus Andika, Liying Sun

**Affiliations:** 1 State Key Laboratory of Crop Stress Biology for Arid Areas and College of Plant Protection, Northwest A&F University 12469, Yangling, China; 2 Guangxi Subtropical Crops Research Institute, Guangxi Academy of Agricultural Sciences, Nanning, China; 3 College of Life Sciences, Northwest A&F University 12469, Yangling, Shaanxi, China; 4 Key Laboratory of Pest Detection and Control in Agriculture and Forestry, College of Agronomy, Xinjiang Agricultural University 117840, Urumqi, Xinjiang, China; 5 Institute of Future Agriculture, Northwest A&F University 12469, Yangling, Shaanxi, China; University of California at Riverside, Riverside, California, USA

**Keywords:** mycovirus, virus variants, viral fitness, transmission, vegetative incompatibility

## Abstract

The high mutation rates of RNA virus replication generate genetically diverse virus variants in infected hosts. However, the effects of mutations on viral fitness and adaptability remain understudied in fungal-virus pathosystems. In this study, a novel hypovirus (single-stranded positive-sense RNA genome, family *Hypoviridae*), designated Valsa pyri hypovirus 1-α (VpHV1-α), and two of its shorter, less prevalent variants (VpHV1-β and VpHV1-γ), that contain different internal deletions in the N-terminal coding region of the viral protein, were identified in phytopathogenic *Valsa pyri* fungal strains. Repeated subculture of a fungal strain infected with VpHV1-α produced VpHV1-β and VpHV1-γ, demonstrating that VpHV1-β and VpHV1-γ were generated by the deletion of the VpHV1-α genome. Compared to VpHV1-α and VpHV1-β, VpHV1-γ, which has a larger deletion, attenuated fungal growth and pathogenicity while accumulating to higher levels. However, it exhibited lower vertical transmission efficiency through spores. Intriguingly, unlike VpHV1-α and VpHV1-β, VpHV1-γ showed restricted horizontal transmission via hyphal anastomosis. This restriction was associated with the induction of programmed cell death and transcriptional activation of vegetative incompatibility-related genes upon VpHV1-γ infection. Additionally, VpHV1-γ infection upregulated key components of RNA silencing (*AGO3* and *DCL2*). Our results reveal a trade-off between viral accumulation and transmission efficiency, influencing the spread and persistence of VpHV1 variants in fungal populations. These findings provide new insights into viral evolution and host adaptation in natural fungal-virus ecosystems.

**IMPORTANCE:**

Studies on mycoviruses are significant for advancing our understanding of viral evolution and host-pathogen interactions. In this study, we identified and characterized a novel hypovirus (VpHV1) infecting the plant-pathogenic fungus *Valsa pyri*. VpHV1 exists as three viral variants (α, β, and γ). Notably, the γ variant, the least prevalent and shortest due to an internal genomic deletion, exhibited unique phenotypic traits: enhanced viral accumulation and symptom severity but impaired horizontal and vertical transmission. Intriguingly, infection by the γ variant induces programmed cell death during hyphal anastomosis with an isogenic fungal strain, thereby preventing viral transmission. This vegetative incompatibility-like reaction may represent a previously unknown defense mechanism in filamentous fungi, functioning to restrict viral spread within genetically homogeneous populations. Our findings demonstrate that transmission ability is a critical selective factor in viral evolution and adaptation within host populations.

## INTRODUCTION

RNA viruses, in particular, exhibit high genetic diversity due to their error-prone RNA-dependent RNA polymerases (RdRP), which lack proofreading activity, leading to the accumulation of mutations (1, 2). Additionally, the genomes of RNA viruses can undergo recombination and reassortment events, further contributing to their genetic diversity and evolutionary potential (3, 4). These mechanisms give rise to the generation of viral quasispecies—heterogeneous viral populations within infected hosts—which serve as reservoirs for the emergence of variants with altered phenotypes and host adaptability (5, 6). A thorough understanding of RNA virus evolutionary dynamics is crucial for anticipating and mitigating emerging viral threats.

Fungal viruses, also known as mycoviruses, are ubiquitous throughout the fungal kingdom (7–9). While most mycoviruses are asymptomatic or cryptic in their natural hosts, a growing number have been found to be pathogenic, causing reduced growth, abnormal pigmentation, impaired reproduction, and/or hypovirulence in fungal hosts (10, 11). Due to these effects, mycoviruses show potential as biocontrol agents against plant-pathogenic fungi. However, their practical application is hindered by transmission challenges (11, 12). Most known mycoviruses lack an extracellular phase in their replication cycle and instead rely on intracellular transmission. They are transmitted intracellularly within the infected fungus during cell division and growth, vertically during asexual and/or sexual spore production, and horizontally between fungal strains through hyphal anastomosis (8, 13). Vegetative compatibility is necessary for the hyphae from different fungal strains or species to fuse (14). Fungal vegetative compatibility is regulated by the vegetative incompatibility (*vic*) genes, which will trigger programmed cell death (PCD) of the hyphal fusion cells when contact between incompatible fungi occurs (15–17). Interestingly, a mycovirus can subvert the induction of PCD to allow its transmission to vegetative incompatible strains (18).

Viruses in the family *Hypoviridae* (order *Durnavirales*) possess a capsidless, single-stranded, positive-sense RNA genome ranging from 7.3 to 18.3 kb. Their genomes typically contain either a single long open-reading frame (ORF) or two ORFs, which encode polyproteins with an N-terminal cis-acting papain-like cysteine protease. To date, hypoviruses have been identified exclusively in fungi and are currently classified into eight genera (19). Although an increasing number of hypoviruses have been discovered in diverse fungal hosts, most molecular insights into hypovirus virology, including replication (20, 21), protein expression (22–24), pathogenesis (25, 26), and host defenses (27–29), have been derived from studies on Cryphonectria parasitica hypovirus 1 (CHV1, genus *Alphahypovirus*), a hypovirulence-inducing virus isolated from the phytopathogenic fungus *Cryphonectria parasitica* (19, 30, 31). Notably, several hypoviruses have been found to be accompanied by defective RNAs, shorter versions of viral RNA segments containing internal deletions (32–37). However, the significance of these defective RNAs for viral replication and host adaptability remains unclear. In *C. parasitica*, DCL2, a key antiviral RNA silencing component, is required for the generation of defective RNA of CHV1 (38).

In this study, we discovered a novel hypovirus, Valsa pyri hypovirus 1 (VpHV1), in the pathogenic fungus *Valsa pyri*. Among different *V. pyri* strains, VpHV1 exists as three viral variants: α, β, and γ. VpHV1-β and -γ are the shorter variants derived from VpHV1-α due to internal deletions. Compared to VpHV1-α and -β, VpHV1-γ, the shortest variant, accumulates at higher levels but exhibited impaired horizontal and vertical transmissions. Our findings further emphasize transmission ability as a critical selective factor in viral evolution and adaptation within natural fungal populations.

## RESULTS

### Identification of a novel hypovirus that exists as three viral variants in *V. pyri* strains

Valsa canker disease, commonly caused by phytopathogenic fungi of the *Valsa* spp. (order Diaporthales) (39, 40), seriously affects pome fruit trees in China’s Xinjiang Province (41, 42). To investigate the fungal species associated with Valsa canker disease in the Xinjiang region, a large number of fungal strains were isolated from Valsa canker disease tissue (tree barks) obtained from various pome fruit trees grown in Changji, Aksu, and Korla cities (Table S1). Most of the isolated fungal strains belonged to *Valsa* spp., with *V. germanica* being predominant in Changji City, while *V. pyri* was the only *Valsa* species isolated from Aksu and Korla cities. For viromic analysis, the isolated *Valsa* fungal strains were screened for the presence of double-stranded RNAs (dsRNAs; Table S1), and the dsRNA samples were subjected to next-generation sequencing (NGS) and bioinformatic analyses. Numerous sequence contigs corresponding to novel virus candidates were identified from the NGS data sets.

**Fig 1:**
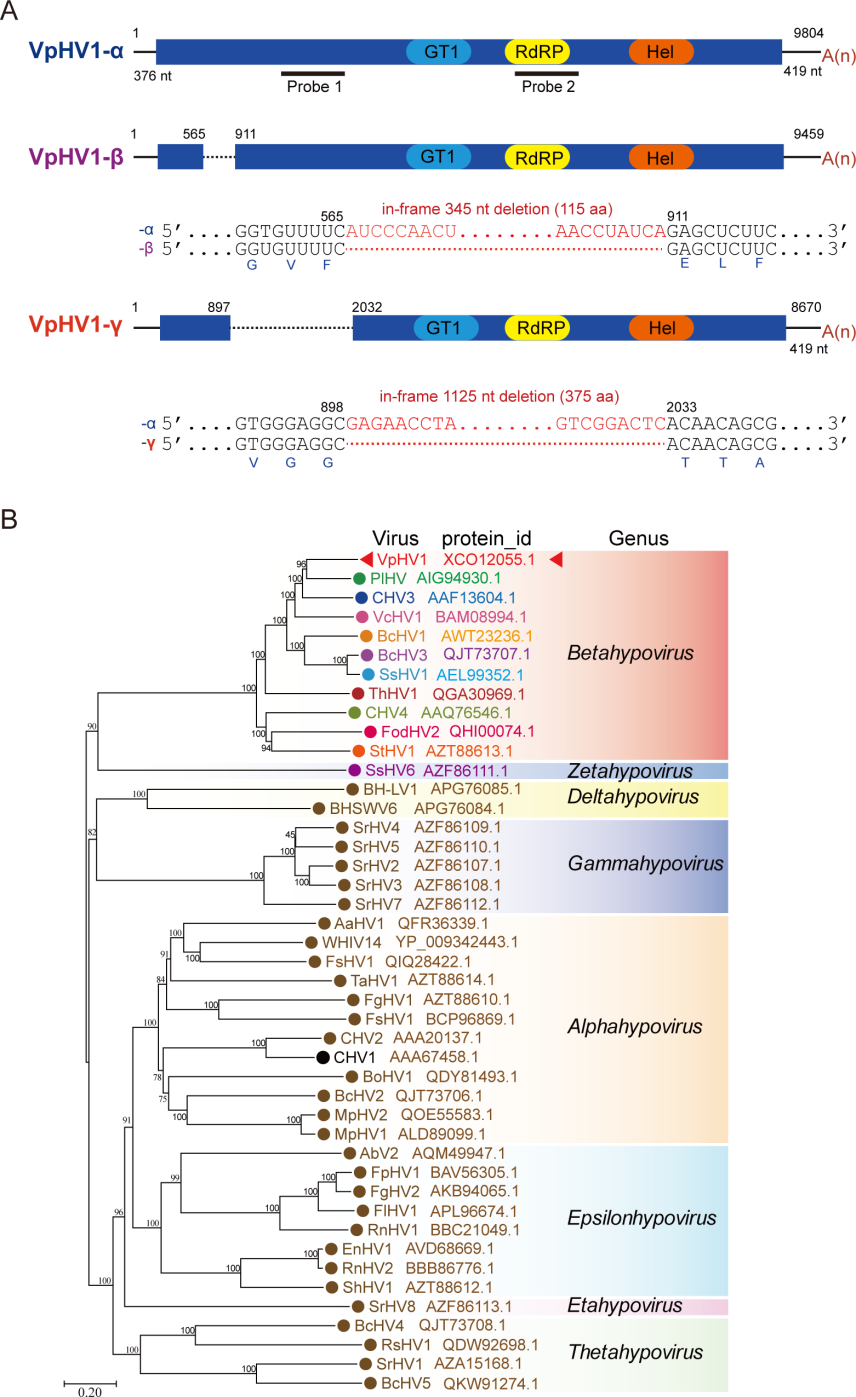
Genome structure and taxonomy of VpHV1. (**A**) Schematic representation (not to scale) of the genome structure of three VpHV1 variants (α, β, and γ). Colored boxes represent ORFs, and black lines represent 5′- and 3′-untranslated regions (UTRs). Nucleotide positions of ORFs and UTRs are indicated. Dashed lines indicate internal genomic deletion regions in VpHV1-β and -γ. The surrounding sequences and lengths of the deleted genomic regions in VpHV1-β and -γ are presented below the genome diagram. The relative positions of conserved domains in the encoded protein are shown in each ORF (colored capsule-shaped forms): glycosyltransferase family 1 (GT1), RdRP, and helicase (Hel). The positions of probes used for RNA blotting are indicated. (**B**) Phylogenetic relationships of VpHV1 to other hypoviruses. The phylogenetic tree is based on a multiple sequence alignment of the RdRP domain. The tree was constructed using the maximum-likelihood method with the best-fit model (LG + F + I + G4). Branch numbers indicate the percentage of trees in which the associated taxa clustered together. The tree is drawn to scale, with branch lengths measured as the number of substitutions per site. Virus names are followed by their accession numbers.

In this study, we focused particularly on two sequence contigs (9,820 and 8,277 nts) that showed homology to hypoviruses based on the BLAST results. Sequence analysis indicated that these two contigs are highly similar, except that the shorter contig appeared to contain internal deletions in the N-terminal coding region of the encoded protein. This virus was tentatively named VpHV1. Screening of numerous fungal strains by RT-PCR using primers flanking the deleted region revealed three different-sized amplification products that varied among fungal strains (Fig. S1), indicating the presence of three VpHV1 variants, hereafter referred to as VpHV1-α, -β, and -γ (ordered from largest to smallest). VpHV1 was not detected in *V. germanica* strains (20 tested) from Changji samples, whereas 50% of *V. pyri* strains (103 tested) carried VpHV1 (Fig. S2). Among these, VpHV1-α was the most prevalent (70%), followed by γ, β, or α + β variants (20%, 6%, and 4%, respectively). Co-culture of several VpHV1-infected strains showed that VpHV1 distributed in vegetatively incompatible strains (Fig. S2). Three *V. pyri* strains (SYD-3-4, SYD-3-8, and HJL-1-22), each harboring a different-sized VpHV1 sequence, were selected for complete viral genome sequencing using RT-PCR and 5′/3′ RACE method. The full-length genomes of VpHV1-α, -β, and -γ are 9,804, 9,459, and 8,670 nt long, excluding the 3′ poly(A) tail), each containing a single large ORF (Fig. 1A). All variants shared identical sequences except that, compared to VpHV1-α, the β and γ variants have in-frame deletions of 345 and 1,125 nt, respectively, in the N-terminal coding region of the encoded protein (Fig. 1A).

The large protein encoded by VpHV1 contained domains characteristic of hypoviridae RdRP, helicase, and glycosyltransferase family 1 (GT1; Fig. 1A). TBLASTN analysis indicated that VpHV1 is most closely related to members of the genus *Betahypovirus*, including Phomopsis longicolla hypovirus 1 (43), Cryphonectria hypovirus 3 (44), and Valsa ceratosperma hypovirus 1 (45), sharing 69%–75% sequence identities (with 87%–91% coverage). Accordingly, phylogenetic analysis grouped VpHV1 with established members of the genus *Betahypovirus* (Fig. 1B).

### VpHV1-γ showed elevated symptoms and accumulation levels in the fungal host

**Fig 2:**
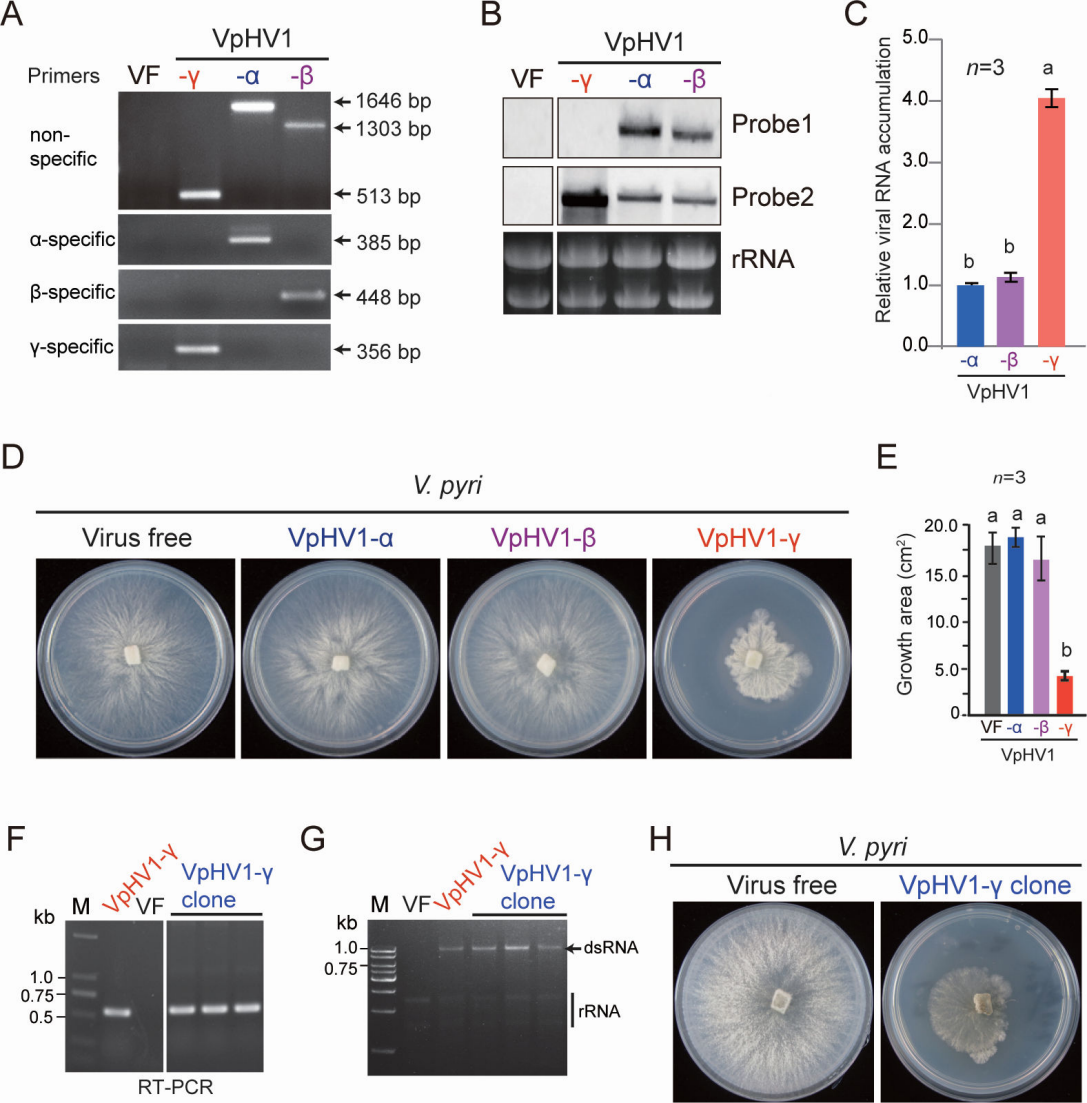
Accumulation and phenotypes of VpHV1 variants in fungal and plant hosts. (**A**) RT-PCR detection of VpHV1 variants using primer sets to confirm independent replication of each variant in *V. pyri*. Nucleotide positions of the amplified viral genome corresponding to VpHV1-α are presented. (**B**) RNA blot analysis of VpHV1 genome accumulation. The relative positions of probes in the VpHV1 genome are indicated in Fig. 1A. (**C**) Relative genome accumulation of VpHV1 variants analyzed by RT-qPCR. Data represent mean ± SD (*n* = 3). Different letters indicate significant differences (*P* < 0.05, one-way analysis of variance [ANOVA]). (**D**) Phenotypic growth and colony morphology of isogenic *V. pyri* strains infected with VpHV1 variants on PDA medium (60 mm plate). Fungi were photographed at 3 days after culturing. (**E**) Colony growth area measured from the experiment described in panel **D**. (**F**) RT-PCR detection of VpHV1-γ derived from an infectious cDNA clone in *V. pyri*. (**G**) dsRNA analysis of VpHV1-γ derived from an infectious cDNA clone. (**H**) Phenotypic growth and colony morphology of *V. pyri* strains infected with VpHV1-γ derived from an infectious cDNA clone on PDA medium (60 mm plate).

The SYD-3–4, SYD-3–8, and HJL-1–22 strains harbored VpHV1-α, -β, and -γ, respectively. We obtained a virus-free strain through single-spore isolation of HJL-1–22 strain. To introduce all VpHV1 variants into the same isogenic background, VpHV1-α and -β were then transferred into a virus-free strain that was derived from single-spore isolation of HJL-1–22 strain via hyphal fusion with the SYD-3–4 and SYD-3–8 strains (Fig. S3). RNA-seq using the ribosome-depleted total RNA confirmed that no other viruses were present in the virus-free HJL-1-22 strain and the VpHV1 variants-infected strains derived from hyphal fusion (File S1). Additional RT-PCR detection and reverse transcription-quantitative PCR (RT-qPCR) analysis of fungal strains using primer sets specific for each strain designed based on the deleted region and specific sequences in VpHV1-β and -γ variants (Fig. S4A) further confirmed single infection of each variant (Fig. 2A; Fig. S4B), indicating that all variants can independently replicate in the fungal host. RNA blotting and RT-qPCR analyses revealed similar accumulation levels of VpHV1-α and -β, whereas VpHV1-γ accumulated at markedly higher levels than the other two variants (Fig. 2B and C). Notably, infection with VpHV1-α and -β did not affect the growth and morphology of the fungal host on potato dextrose agar (PDA) medium, whereas infection with VpHV1-γ severely inhibited host growth (Fig. 2D and E). In an attempt to generate the infectious cDNA clones of all VpHV1 variants, only the construction of the VpHV1-γ clone was successful. Transformation of a virus-free strain with a fungal promoter-driven γ variant clone resulted in virus replication as confirmed by RT-PCR detection and viral dsRNA accumulation (Fig. 2F and G) and coincided with reduced fungal growth (Fig. 2H). These results further confirm the replicative independence and strong pathogenicity of VpHV1-γ.

### Proteolytic processing of the N-terminal region of the polyprotein of VpHV1 variants

**Fig 3:**
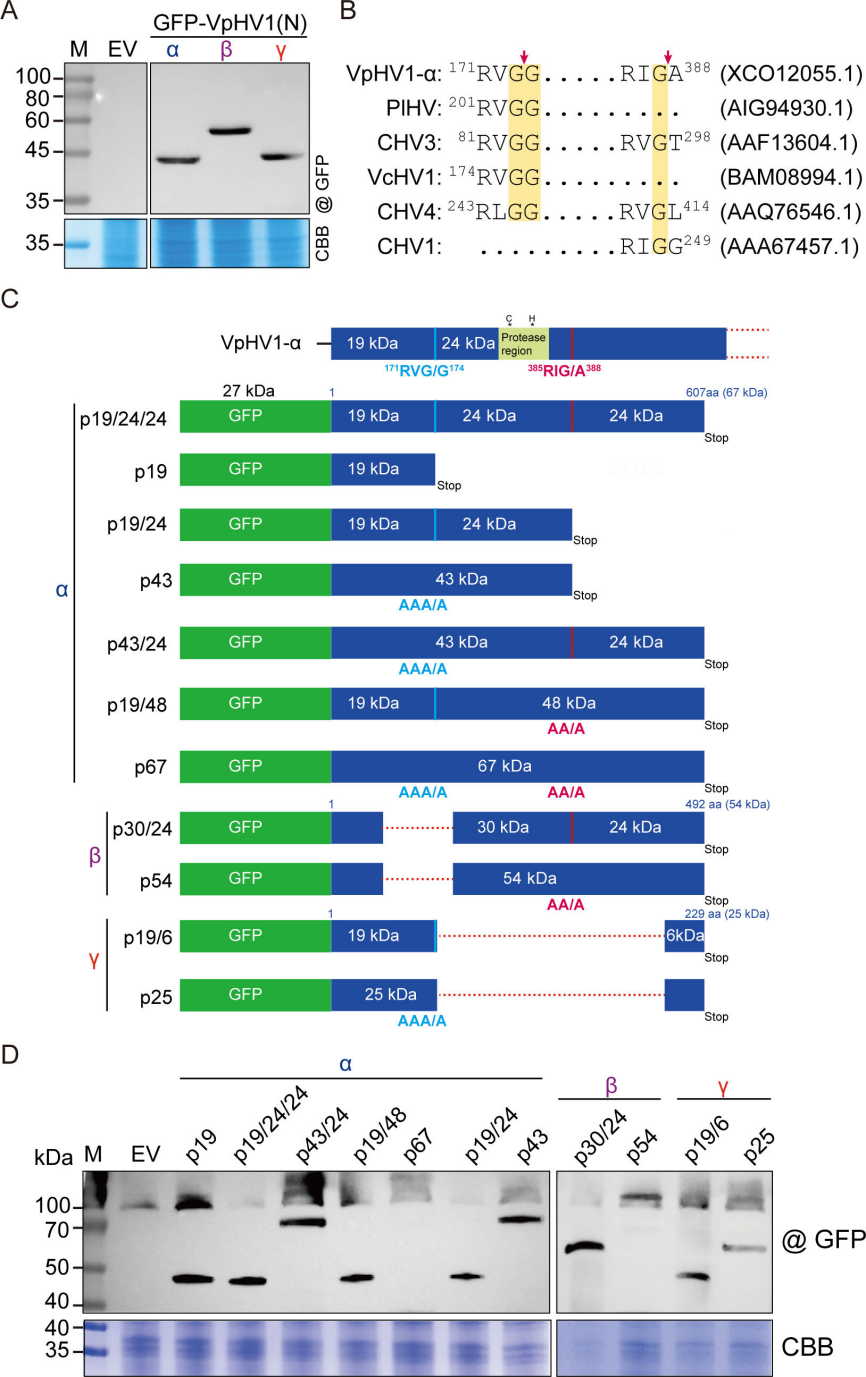
Proteolytic cleavage sites in the N-terminal region of the polyprotein of VpHV1 variants. (**A**) Western blot analysis of the expression of the N-terminal portion of polyproteins encoded by VpHV1 variants fused with GFP in insect cells. EV, empty vector. (**B**) Putative proteolytic cleavage sites conserved in the N-terminal region of the polyprotein of VpHV1 and other hypoviruses. Red arrows indicate the position of peptide bond cleavage. GenBank accession numbers are presented inside the parentheses. (**C**) Schematic diagram of the DNA constructs used for fusion protein expression in insect cells, based on the coding region sequences of the N-terminal region of the polyprotein of VpHV1 variants. In the VpHV1-γ, cleavage between G^173^−G^174^ and G^387^−A^388^ is predicted to release ~19 kDa and ~24 kDa proteins. A conserved protease domain containing putative catalytic cysteine and histidine residues is shown in a small colored box. In some constructs, amino acids in the RVGG and RIGA cleavage sites were substituted with alanine. (**D**) Western blot analysis of fusion protein expression in insect cells using the constructs described in panel **C**.

Hypoviruses encode a papain-like cysteine protease at the N-terminal region of their polyprotein (44, 46). To investigate the proteolytic processing of VpHV1 polyproteins, the N-terminal portion of each polyprotein encoded by VpHV1-α, -β, and -γ was fused with GFP, expressed in insect cells, and analyzed by western blotting. For VpHV1-α and -γ, the expression of GFP-fused 67 kDa and 25 kDa polypeptides of the N-terminal portion of the polyproteins, respectively, yielded a protein band of approximately 45 kDa, suggesting the release of an 18–20 kDa polypeptide (after subtracting the 27 kDa GFP tag) from the N-terminal. In contrast, the GFP-fused 54 kDa polypeptide of VpHV1-β produced a band of ~60 kDa, indicating the cleavage of a ~ 27 kDa polypeptide (Fig. 3A).

Sequence alignment of the N-terminal portion of the polyproteins of hypoviruses identified two putative self-cleavage sites in VpHV1-α that are also present in CHV3, CHV4, and CHV1 (44, 46, 47) (Fig. 3B; Fig. S5). The cleavage of putative ^171^RVGG^174^ and ^385^RIGA^388^ sites is predicted to produce 19 and 24 kDa proteins (Fig. 3C). A conserved protease domain containing putative catalytic Cys and His residues (47) is present in the 24 kDa protein. Notably, VpHV1-β lost the ^171^RVGG^174^ cleavage site, whereas this site is still present in the γ variant, suggesting that it is possibly responsible for the release of the 18–20 kDa polypeptide (Fig. 3A).

To confirm the proteolytic processing of these two cleavage sites, alanine substitutions at each or both cleavage sites were introduced to the DNA constructs to express GFP-fused N-terminal protein of VpHV1 variants as described above (Fig. 3C). As expected, western blot analysis showed the consistent pattern indicating that alanine substitution abolished the release of 19 and 24 kDa polypeptides (Fig. 3D). Notably, the proteolytic processing of ^171^RVGG^174^ cleavage site was observed in VpHV1-γ, which lacks the entire 24 kDa coding region (see p19/6 and p25 constructs; Fig. 3C and D). The function of the 19 kDa protein is unclear. Although it may be a protease, this is uncertain as no conserved catalytic domain was identified. Another plausible scenario is that cellular proteases recognize and cleave the ^171^RVGG^174^ site. Meanwhile, the cleavage of the ^385^RIGA^388^ site persisted in VpHV1-β, although it has lost a large portion of 19 kDa coding region (see p30/24 and p54 constructs; Fig. 3C and D). Together, these results suggest that the N-terminal portion of VpHV1 α polyprotein contains two proteolytic cleavage sites, each responsible for releasing 19 kDa and 24 kDa proteins, whereas VpHV1-β and -γ appear to retain only one of these cleavage sites.

### Emergence of VpHV1-β and -γ from the α variants during fungal subculture

**Fig 4:**
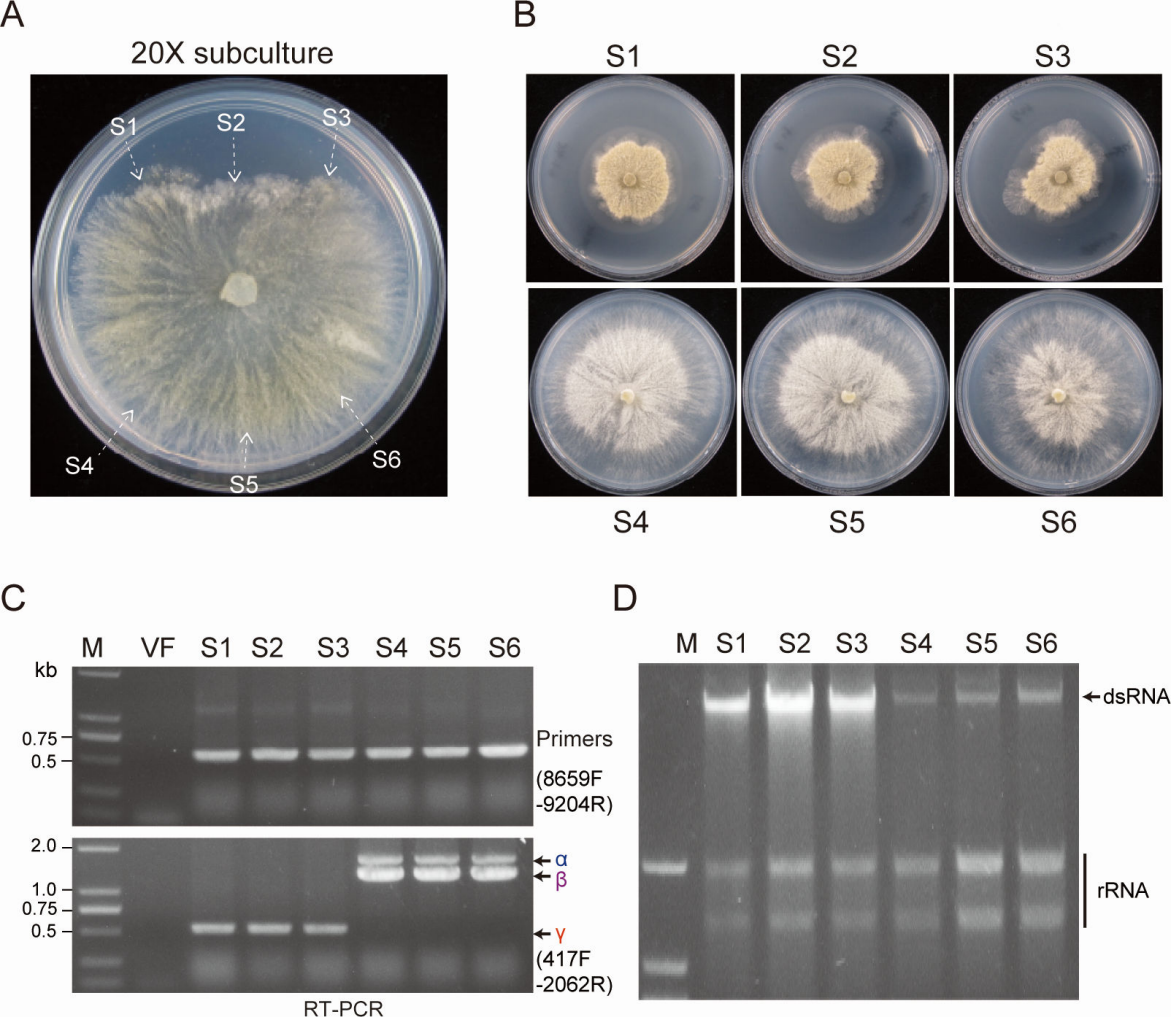
Emergence of VpHV1-β and -γ from α variant during repeated subculturing. (**A**) Colony morphology of VpHV1-α-infected *V. pyri* strain on PDA medium (60 mm plate) after repeated subculturing for 20 times. Fungal colonies were photographed at 3 days after culturing. The colony area where mycelial plugs were obtained for culturing to a fresh PDA medium is indicated (S1−6). (**B**) Phenotypic growth and colony morphology of fungal strains on PDA medium (60 mm plate) derived from different areas described in panel **A**. Fungal colony was photographed at 3 days after culturing. (**C**) RT-PCR detection of VpHV1 variants in fungal strains described in panel **B**. (**D**) Viral dsRNA analysis of fungal strains described in panel **B**.

In previous studies, the defective RNAs of CHV1 and AaHV1 frequently emerged during laboratory fungal culture (32, 38), but it remained unknown whether these defective RNAs could evolve into independently replicating virus variants. To investigate whether VpHV1-β and -γ could arise from VpHV1-α following prolonged infection, we continuously subcultured the VpHV1-α-infected strain in the laboratory. After 20 subcultures on PDA medium, we observed phenotypic change in a portion of the colony edge, where the mycelia grew slowly and became deformed (Fig. 4A; Fig. S1 to S3). This morphology closely resembled that of the VpHV1-γ-infected strain, while the other side still maintained the original phenotype. The mycelia from both deformed and normal areas were picked up and subcultured on a fresh PDA medium for phenotypic observation and RNA extraction.

The fungal strains derived from the deformed colony area showed severe growth restriction compared to those from normal colony area (Fig. 4B). RT-PCR and sequence analysis revealed that VpHV1-γ accumulated in deformed strains, while the normal strains harbored VpHV1-α and -β (Fig. 4C). Additionally, dsRNA analysis showed higher viral dsRNA accumulation in the deformed strains (Fig. 4D), consistent with the elevated levels of VpHV1-γ compared to other variants (Fig. 2B and C). VpHV1-γ obtained from repeated laboratory subcultures remained stable without further changes upon continued subculturing, indicating that this transition was irreversible. These findings demonstrate how VpHV1-β and -γ emerge from internal deletions in VpHV1-α genome during viral replication.

### VpHV1-γ reduces fungal virulence and pycnidia formation

**Fig 5:**
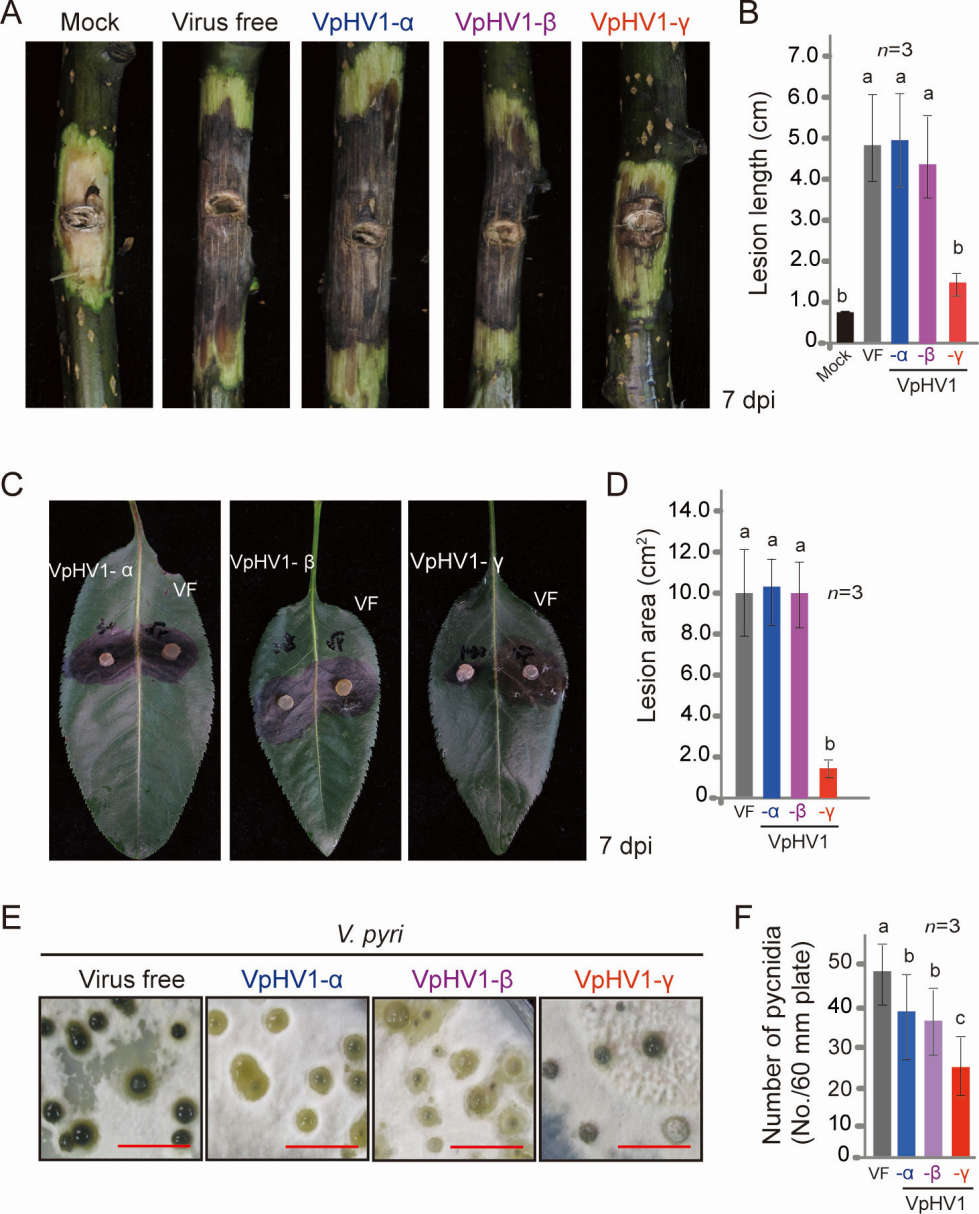
Virulence and pycnidia formation of *V. pyri* strains infected with VpHV1 strains. (**A**) Fungal inoculation assay on pear twigs. Fungal lesions were photographed 7 days after inoculation. (**B**) Lesion length measured on twigs from panel **A**. Data represent mean ± SD (*n* = 3). Different letters indicate significant differences (*P* < 0.05, one-way ANOVA). (**C**) Fungal inoculation assay on pear leaves. Lesions were photographed 7 days after inoculation. (**D**) Lesion area measured on leaves from panel **C**. (**E**) Pycnidia formation in *V. pyri* cultured on PDA medium (90 mm plate) after 30 days. Scale bars: 1 cm. (**F**) Number of mature pycnidia per fungal colony (from panel **E**).

To examine the effect of VpHV1 infection on the pathogenicity of *V. pyri*, we inoculated virus-free and VpHV1-infected strains on the detached branches and leaves of pear trees and measured fungal lesion size. The inoculation assay revealed that strains infected with VpHV1-α and -β produced lesions of similar size to those of the virus-free strain on both branches and leaves. In contrast, the VpHV1-γ-infected strain formed significantly smaller lesions than the other three strains (Fig. 5A through D), indicating that only VpHV1-γ reduces fungal virulence, which is related to its inhibition of fungal growth (Fig. 2D and E). Further observation showed that infection with all VpHV1 variants reduced pycnidia formation, with VpHV1-γ exhibiting a more pronounced effect than VpHV1-α and -β (Fig. 5E and F).

### VpHV1-γ is impaired in horizontal and vertical transmission

While VpHV1-α and -β were observed to transmit to vegetatively compatible virus-free strains through hyphal anastomosis (Fig. S3), it was unexpected that co-culturing VpHV1-γ-infected strain with an isogenic virus-free strain resulted in PCD at the hyphal contact area, which appeared to hinder virus horizontal transmission (Fig. S3). To further investigate this phenomenon in detail, VpHV1-free and VpHV1-infected strains were transformed to carry a hygromycin B (Hyg) resistance marker and used for co-culturing experiment. In these assays, horizontal transmission of VpHV1-α and -β to Hyg-tagged virus-free strain was confirmed, as no PCD occurred. Similarly, bidirectional transmissions of viral variants were observed when the VpHV1-α-infected strain and Hyg-tagged VpHV1-β-infected strain were co-cultured (Fig. S6).

**Fig 6:**
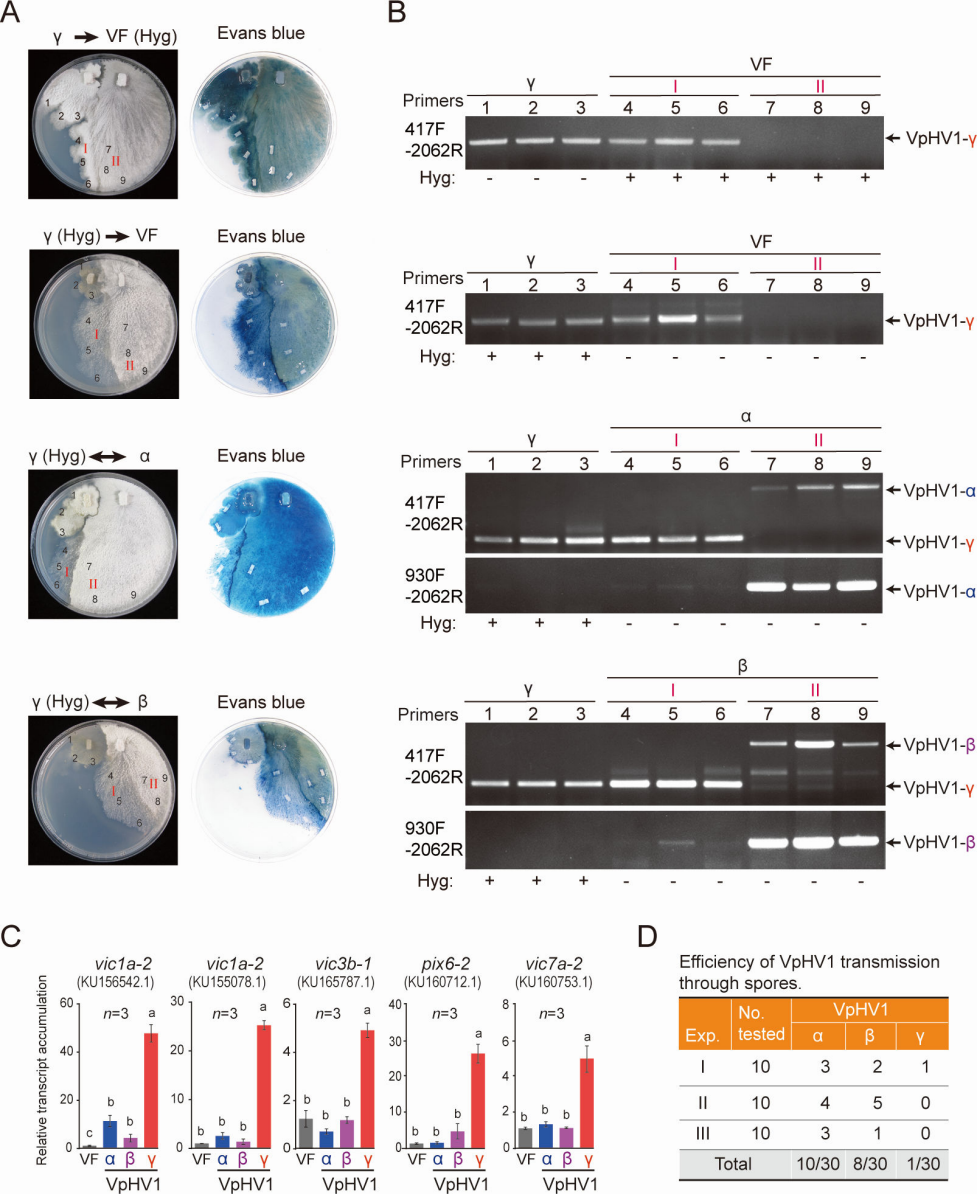
Impaired horizontal and vertical transmission of VpHV1-γ. (**A**) Co-culture of γ variant-infected *V. pyri* strains with isogenic virus-free and VpHV1-α or -β-infected strains on PDA medium (90 mm plate). Fungal colonies were photographed at 7 days after co-culturing. “Hyg” indicates the fungal strain tagged with a hygromycin B resistance marker. The colony areas (1–9) from which mycelial plugs were obtained for transfer to fresh PDA medium are indicated. Regions “I” and “II” mark colony areas where hyphal fusion occurred before and after PCD induction, respectively. Fungal colonies were stained with Evans blue dye to visualize dead cells. (**B**) RT-PCR detection of VpHV1 variants in fungal strains from the experiment described in panel A. Nucleotide positions of the amplified viral genome corresponding to VpHV1-α are presented. Fungal strains’ sensitivity (−) or resistance (+) to hygromycin (Hyg) is indicated. (**C**) Relative transcript accumulation of *vic* genes in *V. pyri* strains infected with VpHV1 variants. Data represent mean ± SD (*n* = 3). Different letters indicate significant differences (*P* < 0.05, one-way ANOVA). (**D**) Summary of RT-PCR detection results for VpHV1 variant transmission through spores.

When the VpHV1-γ-infected strain and Hyg-tagged virus-free strain or Hyg-tagged VpHV1-γ-infected strain and virus-free strain were co-cultured, a bordering area (a line of barrage) indicative of PCD was developed (Fig. 6A, staining with Evans blue dye to visualize dead cells). However, colony regions where hyphal fusion occurred prior to cell death induction were also observed (Fig. 6A, a thin mycelium area was marked as region I). Fungal culturing and RT-PCR analysis confirmed this observation based on hygromycin resistance/sensitivity (Fig. S7) and the presence of VpHV1-γ (Fig. 6B, region I). Notably, RT-PCR verified that VpHV1-γ was not transmitted beyond the PCD border (Fig. 6A and B, a dense mycelium area was marked as region II). Thus, unlike the immediate PCD typically induced between vegetatively incompatible strains, PCD triggered by the VpHV1-γ was delayed, occurring only after initial hyphal fusion. A similar delayed PCD, which blocks horizontal virus transmission, was observed when the hyg-tagged VpHV1-γ-infected strain was co-cultured with the VpHV1-α or -β-infected strains (Fig. 6A and B, region II). Intriguingly, in fungi derived from the initial hyphal fusion zone (region I), where the γ and α/β variants co-infected the same host, the presence of VpHV1-γ was associated with significantly reduced accumulation of VpHV1-α and -β (Fig. 6B, region I). This suggests that VpHV1-γ exerts antagonistic effects against VpHV1-α and -β.

Next, we want to gain insights into the molecular mechanism underlying PCD induction associated with VpHV1-γ infection. Genetic studies in *C. parasitica* have identified several genes in *vic* genetic loci that contribute to restricting the horizontal transmission of CHV1 (48, 49). Four of these genes, *vic1a-2* (encoding a protein with HET domain), *vic3b-1* (a Life-guard–like protein), *pix6-2* (containing a DUF 1040 domain), and *vic7a-2* (an Ankyrin repeat protein), were simultaneously disrupted to generate quadruple *vic* mutant strains capable of transmitting hypoviruses into divergent *vic* genotypes (50, 51). Given the evolutionary closeness between the genera *Cryphonectria* and *Valsa* (order Diaporthales) and genetic analyses suggesting that the *C. parasitica vic* system resembles the nonself recognition systems in other fungi (48), we used RT-qPCR to analyze the relative transcript levels of the homologs of these four genes in *V. pyri* (Table S2) during infection with different VpHV1 variants. The results revealed that the transcript levels of these *vic* genes were significantly elevated exclusively during VpHV1-γ infection (Fig. 6C). These findings suggest that impaired horizontal transmission of VpHV1-γ is linked to the transcriptional activation of *vic* genes.

Vertical transmission is also crucial for the distribution of fungal viruses in nature. To assess vertical transmission of VpHV1 variants, we performed single-spore isolation followed by RT-PCR detection. The results revealed that the transmission efficiency of the γ variant was significantly lower (3.3%) than that of VpHV1-α and -β (27%–33%; Fig. 6D).

### VpHV1-γ is targeted by antiviral RNA silencing

**Fig 7:**
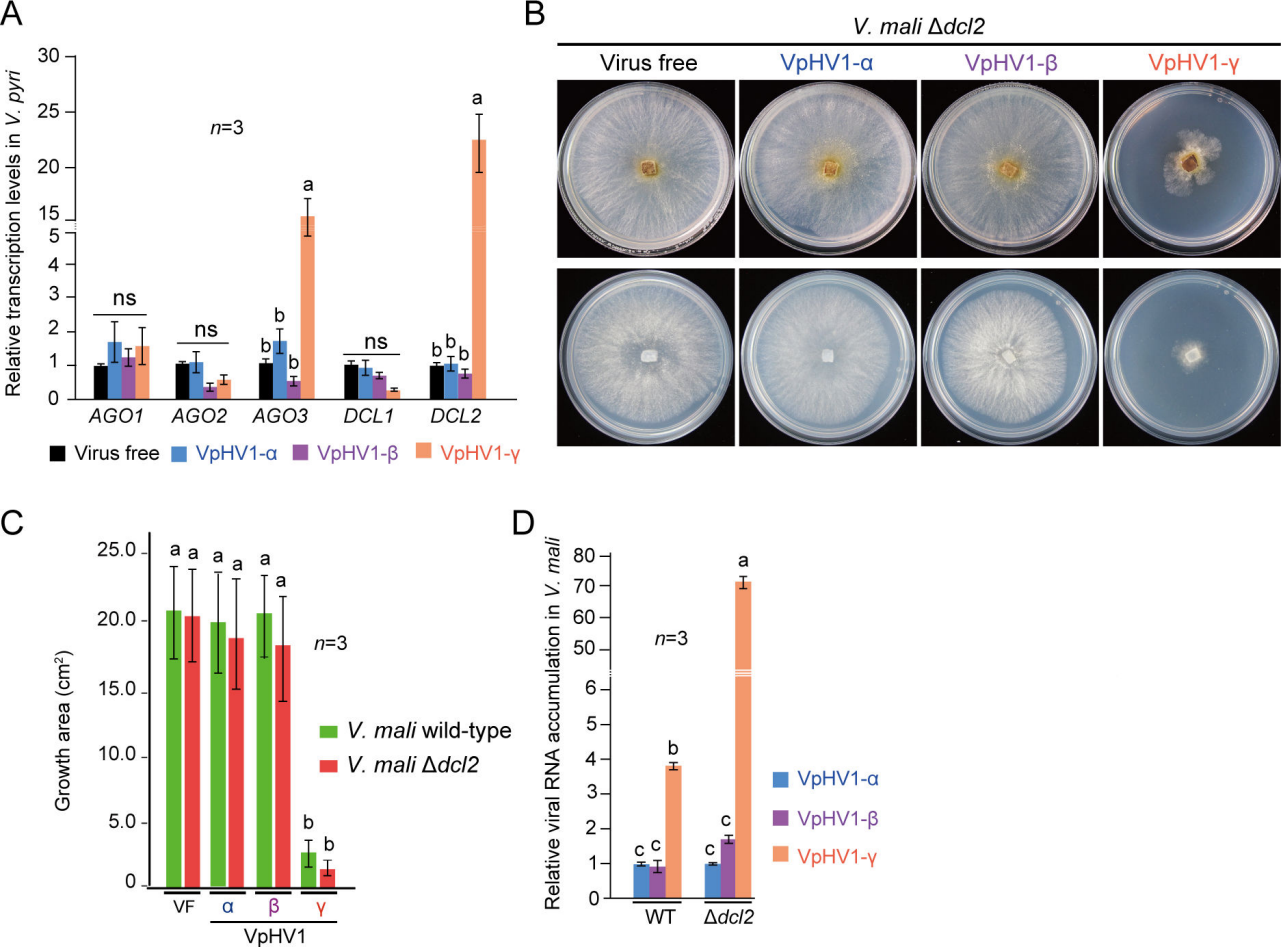
Induction of RNA silencing by VpHV1-γ infection. (**A**) Relative transcript accumulation of *DCL* and *AGL* genes in *V. pyri* strains infected with VpHV1 variants. Data represent mean ± SD (*n* = 3). Different letters indicate significant differences (*P* < 0.05, one-way ANOVA). (**B**) Phenotypic growth and colony morphology of wild-type and *DCL2* knockout mutant (Δ*dcl2*) *V. mali* strains infected with VpHV1 variants on PDA medium (60 mm plate). Fungi were photographed at 3 days after culturing. (**C**) Colony growth area measured from the experiment described in panel **B**. (**D**) Relative genome accumulation of VpHV1 variants in wild-type and Δ*dcl2 V. mali* strains analyzed by RT-qPCR. Data represent mean ± SD (*n* = 3). Different letters indicate significant differences (*P* < 0.05, one-way ANOVA).

RNA silencing plays a major role in antiviral defense in fungi (27, 52, 53). Notably, transcriptional upregulation of RNA silencing key genes such as dicer-like (*DCL*) and AGO-like (*AGL*) genes was observed in various fungal species upon infection by hypoviruses and other mycoviruses (28, 29, 54–57). To determine whether VpHV1 variants also induce RNA silencing, the relative transcript levels of *V. pyri DCL1-2* and *AGL1-3* genes following VpHV1 infection were analyzed using RT-qPCR. The results showed that only VpHV1-γ infection was associated with elevated transcription levels of *AGO3* and *DCL2* genes, whereas the transcription of other genes remained unaffected by all variants (Fig. 7A). This suggests that AGO3 and DCL2 play a crucial antiviral role in *V. pyri*.

Since *V. pyri* mutants with disruption in RNA silencing-related genes are not available, we introduced VpHV1 variants into wild-type and *DCL2* knockout mutant (Δ*dcl2*) of *V. mali* (58) by transfecting *V. mali* protoplasts with total RNA extracted from infected *V. pyri* strains. Infection of VpHV1 variants in *V. mali* strains was confirmed by viral dsRNA analysis (Fig. S8A). Only VpHV1-γ reduced the growth and pathogenicity of both wild-type and Δ*dcl2* strains, with a stronger effect observed in the Δ*dcl2* mutant (Fig. 7C; Fig. S8B and C). RT-qPCR analysis revealed that the accumulation level of VpHV1-γ was significantly higher than that of equally accumulated α and β strains in both wild-type and Δ*dcl2* strains. Moreover, compared to its accumulation in the wild-type strain, only VpHV1-γ showed elevated accumulation in the Δ*dcl2* strain (Fig. 7D). Together, these results demonstrated that VpHV1-γ is recognized and targeted by fungal antiviral RNA silencing.

## DISCUSSION

The discovery of CHV1 in *C. parasitica* is particularly significant in mycovirology as it marked the first observation on hypovirulence induced by the mycovirus infection. Since then, numerous hypoviruses have been identified across various fungal species, many of which confer hypovirulence to their hosts (32, 35, 36, 44, 59, 60). In addition to CHV1, three other hypoviruses, Cryphonectria hypovirus 2–4 (CHV2–CHV4), have been identified in *C. parasitica* (44, 47, 61). While CHV3 and CHV4 belong to the genus *Betahypovirus*, VpHV1 is more closely related to CHV3 (Fig. 1B). Typical of betahypoviruses, CHV3 encodes a polyprotein with a putative N-terminal proteinase predicted to self-cleavage, releasing 32.5 kDa protein (44, 62). CHV3 infection is associated with DI RNAs containing large internal deletions and nucleotide sequence divergences (33). Although many hypoviruses are linked to DI RNAs, suggesting that intermolecular RNA recombination frequently occurs during genome replication due to RdRP errors (63), no independently replicating hypovirus variant with an internal genomic deletion had been identified prior to this. This appears to result from RNA recombination of the hypovirus genome, often causing deletions in coding regions critical for RdRP activity (32, 33, 37).

The viability of VpHV1-β and -γ indicates that the N-terminal portion of VpHV1-α-encoded polyprotein is dispensable for viral replication. These VpHV1 variants, with internal genomic deletions, resemble CHV1 mutants having deletions in the ORF A coding region (CHV1 Δp69), which were generated from an infectious cDNA clone (64). The CHV1 genome contains two large ORFs, designated ORF A and ORF B (34). The 5′-proximal ORF A encodes a polyprotein, p69, which is proteolytically processed into two polypeptides, p29 and p40, by the papain-like protease domain within p29 (65). Although neither p29 nor p40 is essential for viral replication, both contribute to optimal viral RNA accumulation (64). CHV1 p29 associates with the *C. parasitica trans*-Golgi network, and this association likely facilitates the proliferation of vesicles (66, 67). In particular, p29 plays multifunctional roles as a viral symptom determinant (25, 26) and an antiviral RNA silencing suppressor by inhibiting the transcriptional upregulation of *DCL2* and *AGL2* genes upon virus infection (28, 29). Similarly, p24, released from the N-terminal portion of the CHV4 polyprotein, suppresses transcriptional upregulation of the *DCL2* gene (68). Mutational analysis and eukaryotic expression of the N-terminal portion of VpHV1-encoded polyprotein indicated that two self-cleaved proteins, p19 and p24, are released from the polyprotein (Fig. 3). Notably, the viral phenotypes exhibited by VpHV1-β and -γ raise an intriguing question regarding the functions of p19 and p24, which fundamentally deviate from those observed for CHV1 p29 and p40. While VpHV1-β, which lacks the large part of the p19 coding region, displayed a phenotype similar to that of VpHV1-α, the γ variant, which retains p19 but has a larger genomic deletion, including the entire p24 coding region, showed elevated viral accumulation and conferred growth inhibition and hypovirulence to the fungal host (Fig. 2 and 5). Thus, while p19 function remains unclear, p24 and/or the coding RNA regions appear to negatively affect accumulation potential or RNA genome stability of VpHV1 in the fungal host. One possibility is that p24 activates another layer of the antiviral resistance pathway, which is independent of DCL2.

In facing the host’s antiviral immune system, viruses have evolved multiple strategies, including immune evasion (avoiding detection and clearance by the immune system) and immune counteraction (developing specific proteins or strategies to actively inhibit or interfere with host antiviral responses). VpHV1-α and VpHV1-β had relatively low accumulation levels (Fig. 2B and C), and the expression of *DCL1-2* and *AGL1-3* genes remained unchanged during infection (Fig. 7A). It appears that VpHV1-α and VpHV1-β persist and replicate without activating the host’s immune defenses, whereas VpHV1-γ’s high accumulation potential activates RNA silencing responses. Moreover, only the accumulation of VpHV1-γ was elevated in the Δ*dcl2* mutant strain (Fig. 7A and D), indicating that VpHV1-γ is targeted by antiviral RNA silencing. Notably, the reduction of vertical transmission efficiency of VpHV1-γ correlated with the induction of RNA silencing (Fig. 6D). These observations are similar to those in CHV1, where the deletion of p29 resulted in activation of RNA silencing and reduced vertical transmission through conidia (29, 69). These observations suggest another scenario in which VpHV1 p24 acts as an RNA silencing suppressor by inhibiting RNA silencing activation, albeit at the cost of compromising viral accumulation potential. It would be interesting to investigate the mechanisms by which VpHV1 interacts with the host’s antiviral system and to determine whether p19, p24, or the RNA genome sequence plays a role in these interactions.

In filamentous fungi, the PCD associated with vegetative incompatible reaction serves as a fundamental protection mechanism against the transmission of unfavorable or harmful intracellular contents, including mycoviruses (16, 70). Fungal strains possessing identical alleles at specific *vic* loci can usually undergo hyphal anastomosis (71). However, a line of a barrage was formed when the VpHV1-γ-infected strain was co-cultured with an isogenic virus-free strain, preventing further virus transmission (Fig. 6A and B). Thus, VpHV1-γ infection induces a vegetative incompatible-like reaction during hyphal fusion. Notably, VpHV1-γ infection induced the transcriptional upregulation of *vic* genes that contribute to the restriction of horizontal virus transmission in *C. parasitica* (Fig. 6C), implying that VpHV1-γ infection reprograms *vic* gene expressions. However, it is still unclear how VpHV1-γ infection causes such physiological changes. This vegetative incompatibility-like reaction may represent a previously unknown defense mechanism in filamentous fungi that functions to restrict the spread of viruses within genetically homogeneous fungal populations. Indeed, VpHV1 variants are valuable research materials for uncovering this defense mechanism.

The majority of fungal viruses are asymptomatic, a trait that facilitates their persistence in fungal populations. From an evolutionary perspective, this results from long-term co-evolution between viruses and their hosts. Through the subculture experiments, we demonstrated that VpHV1-γ emerged from a genomic deletion of VpHV1-α, evolving from an asymptomatic virus into a highly accumulated, virulent form, indicating enhanced biological fitness. However, VpHV1-γ is less prevalent than VpHV1-α in the fungal population, likely due to its reduced vertical and horizontal transmissions. Thus, the trade-off between diminished accumulation potential and transmission efficiency plays a key role in maintaining genome integrity and shaping the evolution of VpHV1.

## MATERIALS AND METHODS

### Sample collections and fungal strains

Valsa canker disease samples (stems/twigs) were collected from pome fruit trees in orchards located in Changji City Autonomous Prefecture, Aksu City, and Korla City in the Xinjiang Province of China, during the early summer of 2019.

Fungal strains were isolated from the junctions of Valsa canker lesions and healthy tissue using a previously described method (72). The isolates SYD-3–4, SYD-3–8, and HJL-1–22, harboring VpHV1-α, β, and γ, respectively, were maintained as original standard strains. All fungal strains were cultured in PDA medium at 25°C for phenotypic and growth observation. For mycelial collection, strains were grown on PDA medium overlaid with cellophane.

The wild-type and *DCL2*-knockout mutants of *V. mali* strains used in this study were described previously (58).

### RNA isolation, NGS, RT-PCR, and viral genome sequencing

Total RNA and dsRNA were isolated as previously described (55). NGS analyses of the dsRNA-enriched fraction and the ribosome-depleted total RNA were performed on the Illumina HiSeq 4000 platform (Illumina, San Diego, CA, USA) by Hanyu Biotechnology Co., Ltd. (Shanghai, China), following established protocols (73). FastQC (https://www.bioinformatics.babraham.ac.uk/projects/fastqc/) and Trimmomatic (74) were used for data quality control and cleaning of reads, respectively. The Trinity v2.2.0 software (75) was used for *de novo* assembly of the sequence reads. The assembled contigs served as queries for BLASTX searches against viral genome sequences in the NCBI database. HISAT2 (76) was employed to map the reads to the assembled contigs. Samtools (77) was used to count mapped reads for each contig.

For viral genome characterization, first-strand cDNA was synthesized using EasyScript Reverse Transcriptase (TransGen Biotech, China). The quasi-full-length sequence of VpHV1 was amplified by RT-PCR with Kangwei DNA polymerase (China) using virus-specific primers designed from NGS-assembled contigs. The 5′- and 3′-terminal sequences of the VpHV1 genome were verified by RACE (Rapid Amplification of cDNA Ends) (78). All purified PCR products were cloned into the pGEM-T Easy Vector (Promega, USA) and subjected to Sanger sequencing. The primers used in this study are listed in Table S3.

### Construction of baculovirus expression vectors

The coding sequences of N-terminal region of VpHV1 variants-encoded polyprotein (nucleotide position 377–2,197 in VpHV1-α) were amplified by RT-PCR. The DNA fragments were inserted into the binary vector pBIN61-GFP (79), resulting in GFP-N-terminal fusion constructs. Site-directed mutagenesis of putative protease cleavage sites was performed using two PCR steps (80). The fusion constructs were PCR amplified and then inserted into the eukaryotic protein expression vector pQBX, a modified version of pQBD (81). All plasmids generated in this study are described in Table S4.

### Infectious viral cDNA clone, fungal expression constructs, and fungal transformation

The complete genome sequence of VpHV1-γ was amplified using overlapping RT-PCR and inserted into a fungal expression plasmid vector pCPX-HY2 (25), resulting in an infectious VpHV1-γ cDNA clone plasmid.

The preparation of fungal protoplasts and the transfection of plasmid or RNA into fungal protoplasts were carried out as previously described (55, 72).

The methods for sequence analyses, RT-qPCR, RNA blotting, western blotting, pathogenicity assays, protein expression in insect cells, fungal subculture, viral transmission and mycelial compatibility assays, virus inoculation to plants are described in Supplemental Methods.

## Data Availability

The complete viral genome sequence of VpHV1-α was deposited in GenBank (accession no. PP999648.1).

## SUPPLEMENTAL MATERIAL

File S1 mbio.02922-25-s0001.xlsx RNAseq data.

Supplemental Figures mbio.02922-25-s0002.pdf Figures S1 to S8.

Supplemental Text mbio.02922-25-s0003.pdf Supplemental methods.

Supplemental Tables mbio.02922-25-s0004.pdf Tables S1 to S4.

ASM does not own the copyrights to Supplemental Material that may be linked to, or accessed through, an article. The authors have granted ASM a non-exclusive, world-wide license to publish the Supplemental Material files. Please contact the corresponding author directly for reuse.
