# Supplementary material for: Trade-off between accumulation potential and transmission efficiency in hypovirus variants infecting phytopathogenic fungi
Source: mBio. 2026 Jan 21;17(2):e02922-25. doi: 10.1128/mbio.02922-25 (PMC12892957; doi:10.1128/mbio.02922-25)
Supplement: Supplemental Figures — Figures S1 to S8. [file mbio.02922-25-s0002.pdf]

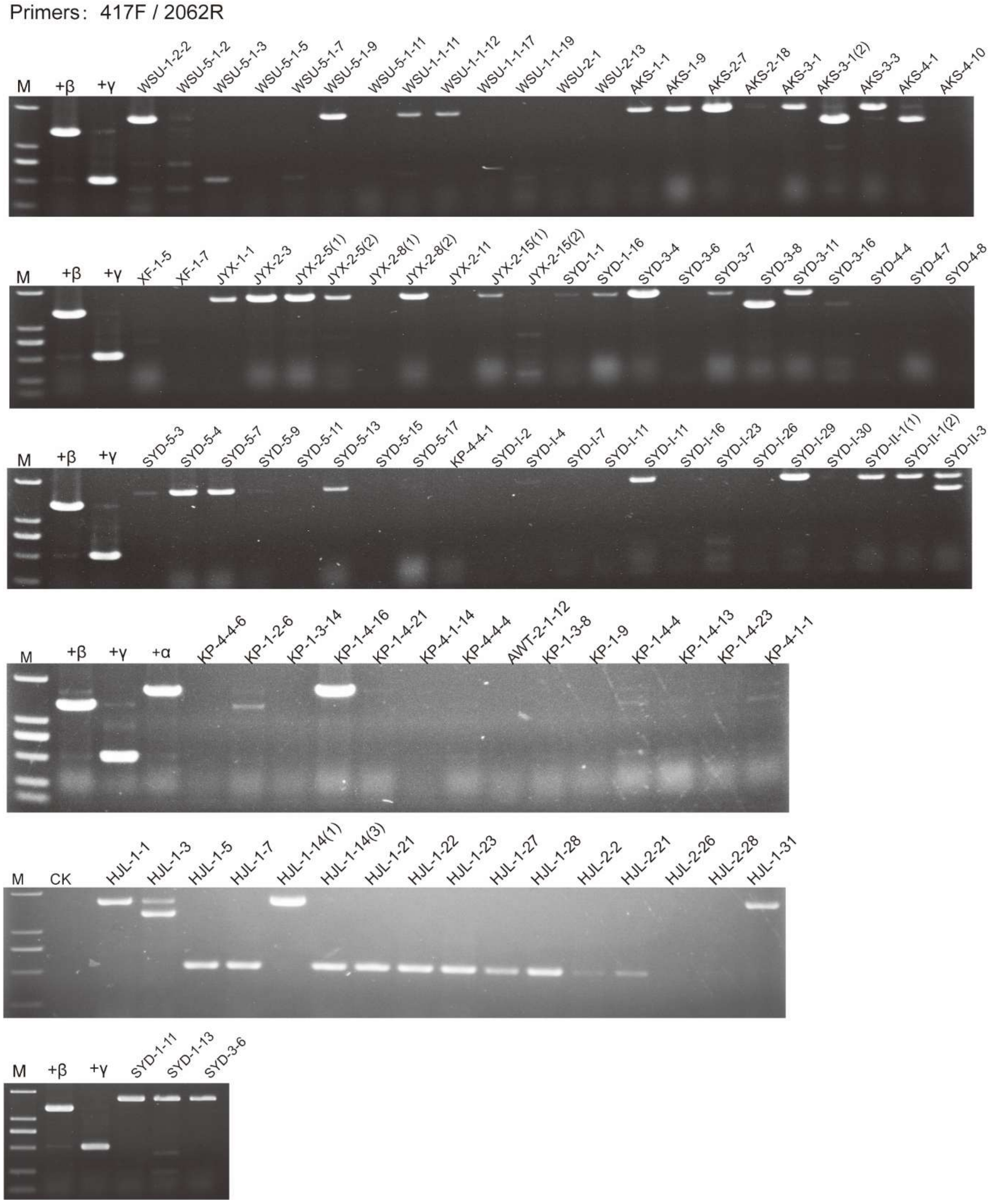

Figure S1. RT-PCR detection of VpHV1 in *V. pyri* strains isolated from pome fruit trees in China's Xinjiang Province.

A

Number of VpHV1-infected fungal strains

Location	α	β	γ	α/β	α/γ	β/γ	α/β/γ	VF	No.tested
Changji	0	0	0	0	0	0	0	20	20
Aksu	10	2	0	0	0	0	0	26	38
Korla	26	1	10	2	0	0	0	26	65
Total	36	3	10	2	0	0	0	66	123

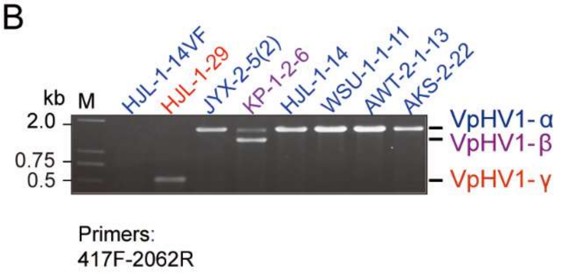

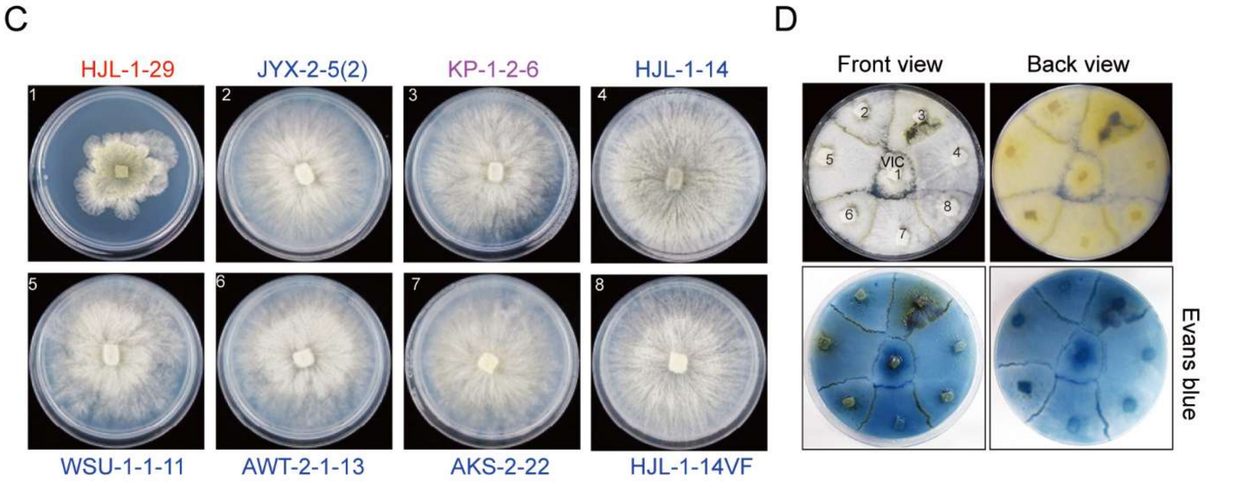

Figure S2. Prevalence of VpHV1 variants in *V. pyri* strains and vegetative incompatibility of VpHV1-infected strains. **A.** Summary of RT-PCR detection results for VpHV1 variants in fungal strains isolated from three different regions in Xinjiang Province. **B.** RT-PCR detection of VpHV1 variants in eight representative fungal strains. **C.** Phenotypic growth and colony morphology of *V. pyri* strains infected with VpHV1 variants on PDA medium (60 mm plate). Fungi were photographed at 7 days after co-culturing. **D.** Co-culture of eight representative fungal strains on PDA medium (90 mm plate) described in panel C. Fungi were photographed at 7 days after culturing. Fungal colonies were stained with Evans blue dye to visualize dead cells.

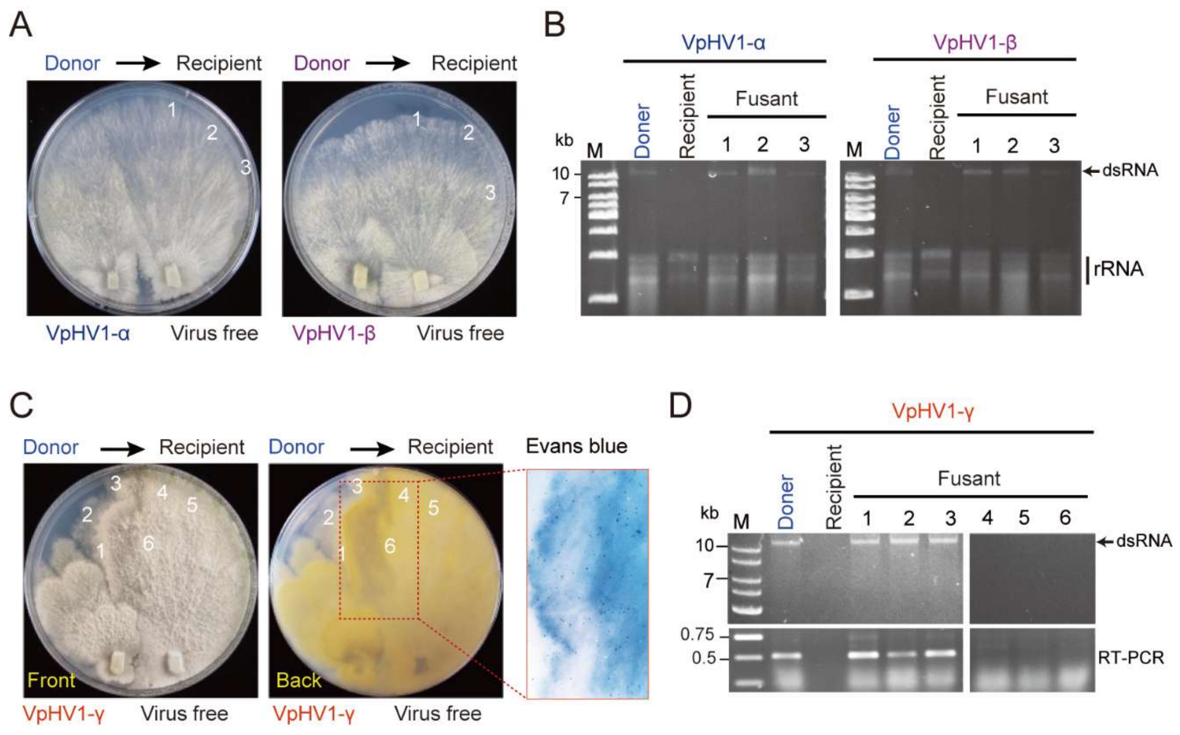

Fig. S3. Transmissibility of VpHV1 variants through hyphal anastomosis. **A.** Co-culture of VpHV1-α or -β variant-infected *V. pyri* strain with an isogenic virus-free strain on PDA medium (90 mm plate). Fungal colonies were photographed at 7 days after culturing. The colony areas (1-3) from which mycelial plugs were obtained for transfer to fresh PDA medium are indicated. **B.** Viral dsRNA accumulation of VpHV1-α and -β variants in fungal strains from the experiment described in panel A. **C.** Co-culture of VpHV1-γ variant-infected *V. pyri* strain with an isogenic virus-free strain on PDA medium (90 mm plate). Fungal colonies were photographed at 7 days after co-culturing. Fungal colonies were stained with Evans blue dye to visualize dead cells. **D.** Viral dsRNA analysis and RT-PCR detection of VpHV1-γ variant in fungal strains from the experiment described in panel C.

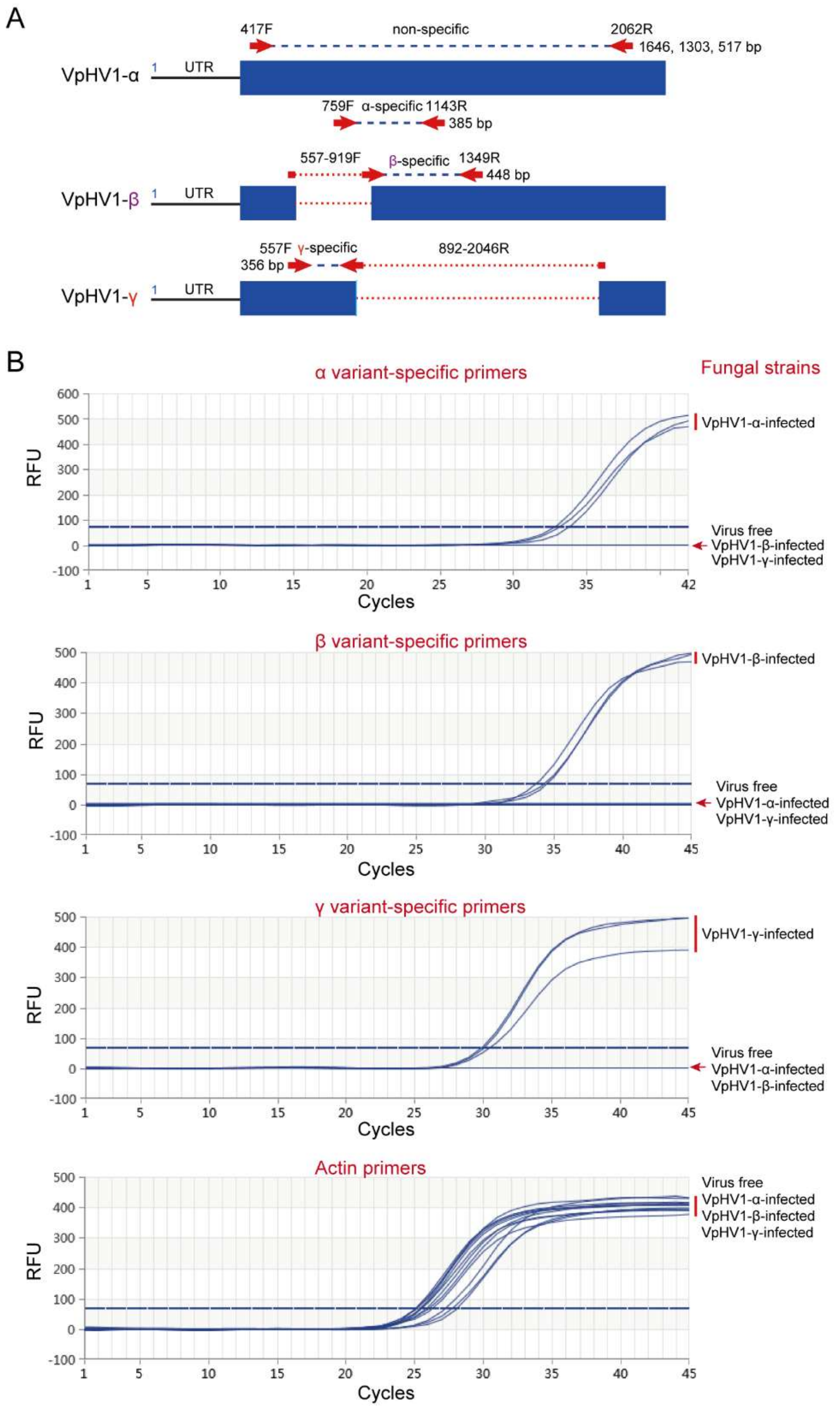

Fig. S4. RT-qPCR detection of VpHV1 variants using primer sets specific to each variant. **A**. Schematic diagram showing nucleotide positions of primers designed based on the deleted region and specific sequences in VpHV1-β and -γ variants. Red arrows indicate primer positions. Dashed blue lines indicate amplified regions. Primer names and PCR product sizes are presented. **B**. Amplification curve (Relative Fluorescence Units [RFU]) plot obtained from RT-qPCR using primers described in A.

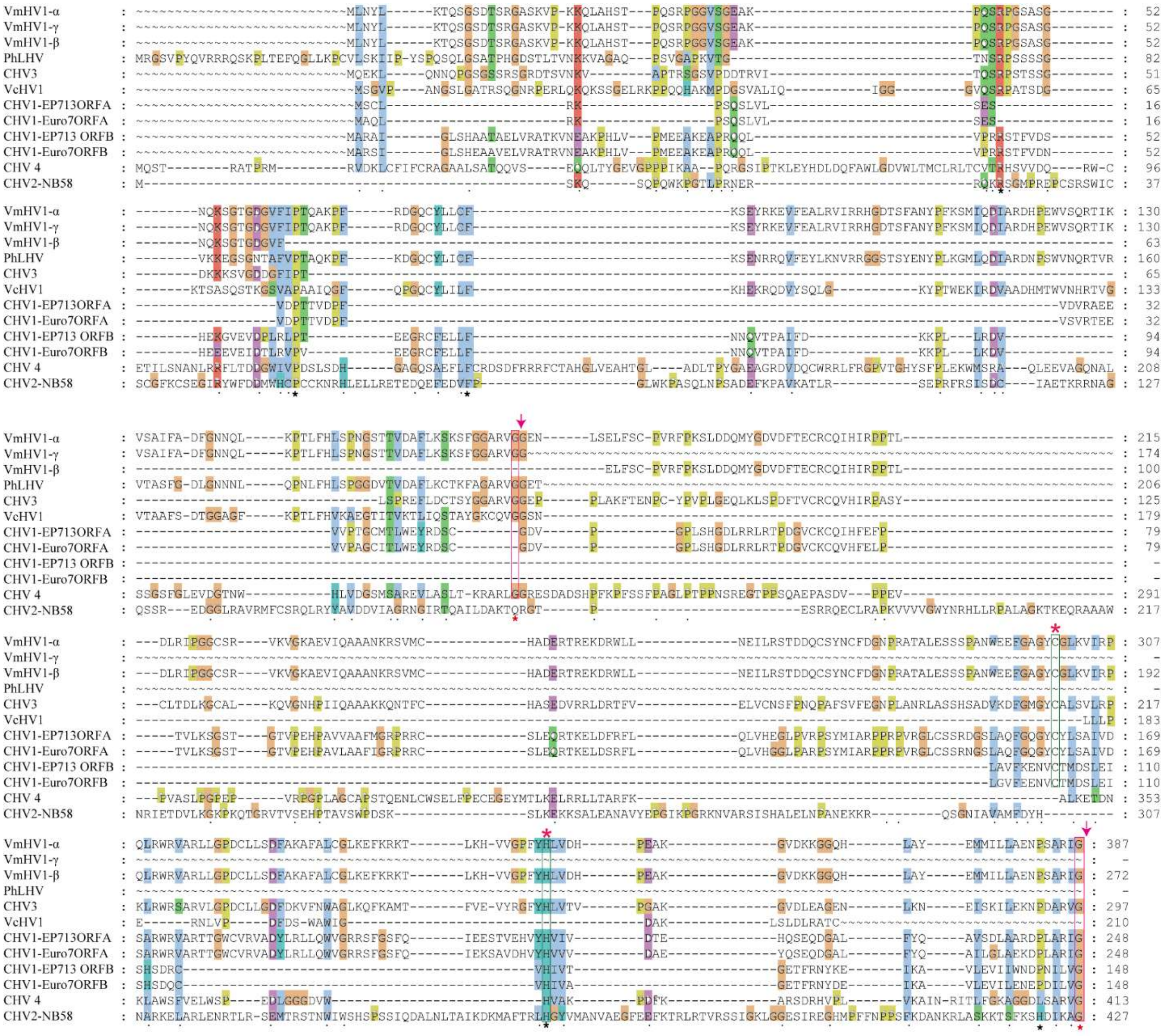

Figure S5. Amino acid sequence alignment of the N-terminal portion of polyproteins encoded by VpHV1 variants and other hypoviruses. Red arrows indicate the position of peptide bond cleavage. Putative catalytic cysteine and histidine residues conserved in protease domain are indicated by red asterisks.

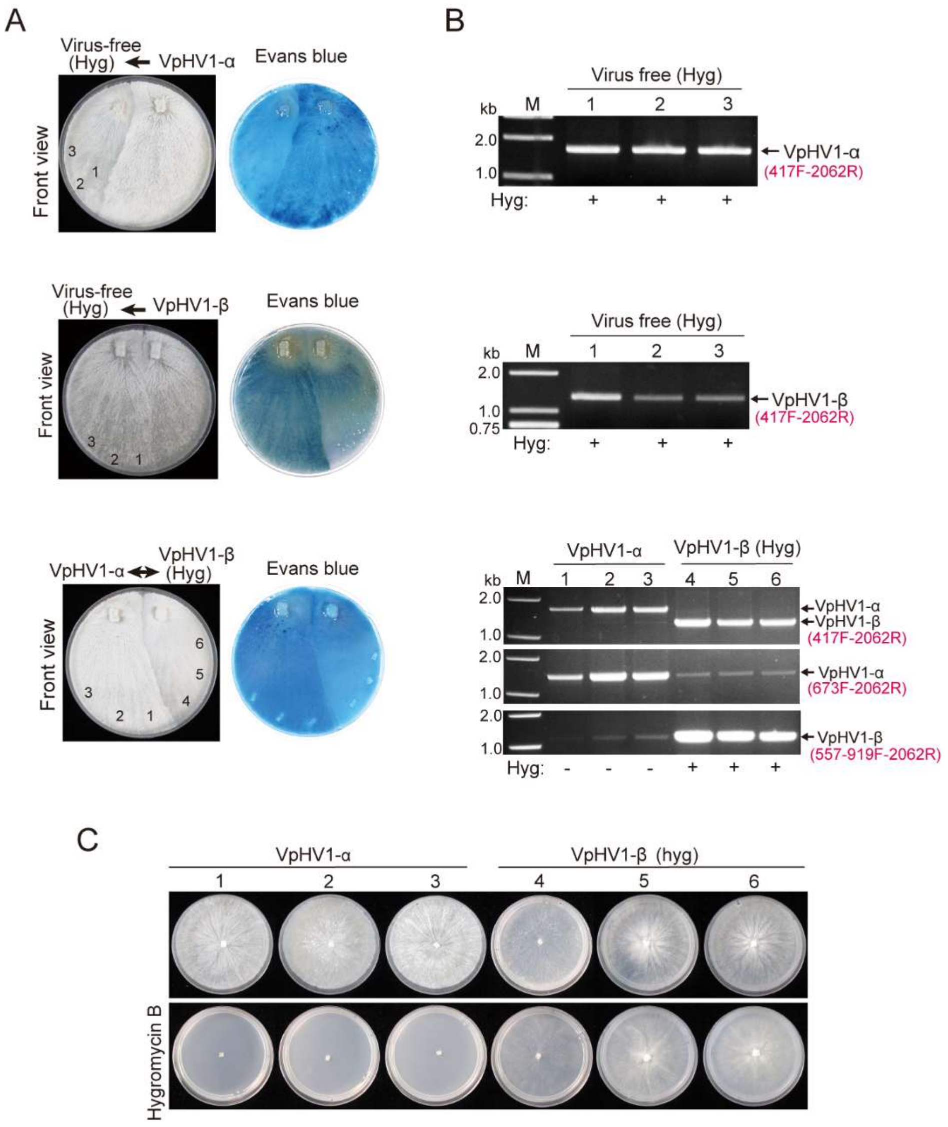

Fig. S6. Transmissibility of VpHV1-α and -β variants through hyphal anastomosis. **A.** Co-culture of VpHV1-α or -β variant-infected *V. pyri* strain with an isogenic virus-free strain and co-culture of VpHV1-α variant-infected strain and VpHV1-β variant-infected strain on PDA medium (90 mm plate). Fungal colonies were photographed at 7 days after co-culturing. "Hyg" indicates the fungal strain tagged with a hygromycin B resistance marker. The colony areas (1-3 and 1-6) from which mycelial plugs were obtained for transfer to fresh PDA medium are indicated. Fungal colonies were stained with Evans blue dye to visualize dead cells. **B.** RT-PCR detection of VpHV1 variants in fungal strains from the experiment described in panel A. Nucleotide positions of the amplified

viral genome corresponding to VpHV1-α variant are presented. Fungal strain sensitivity (-) or resistance (+) to hygromycin (Hyg) is indicated. **C.** Growth of fungal strains from co-culturing VpHV1-α variant-infected strain and VpHV1-β variant-infected strain on PDA medium (60 mm plate) with or without hygromycin B. Fungal colonies were photographed at 3 days after co-culturing.

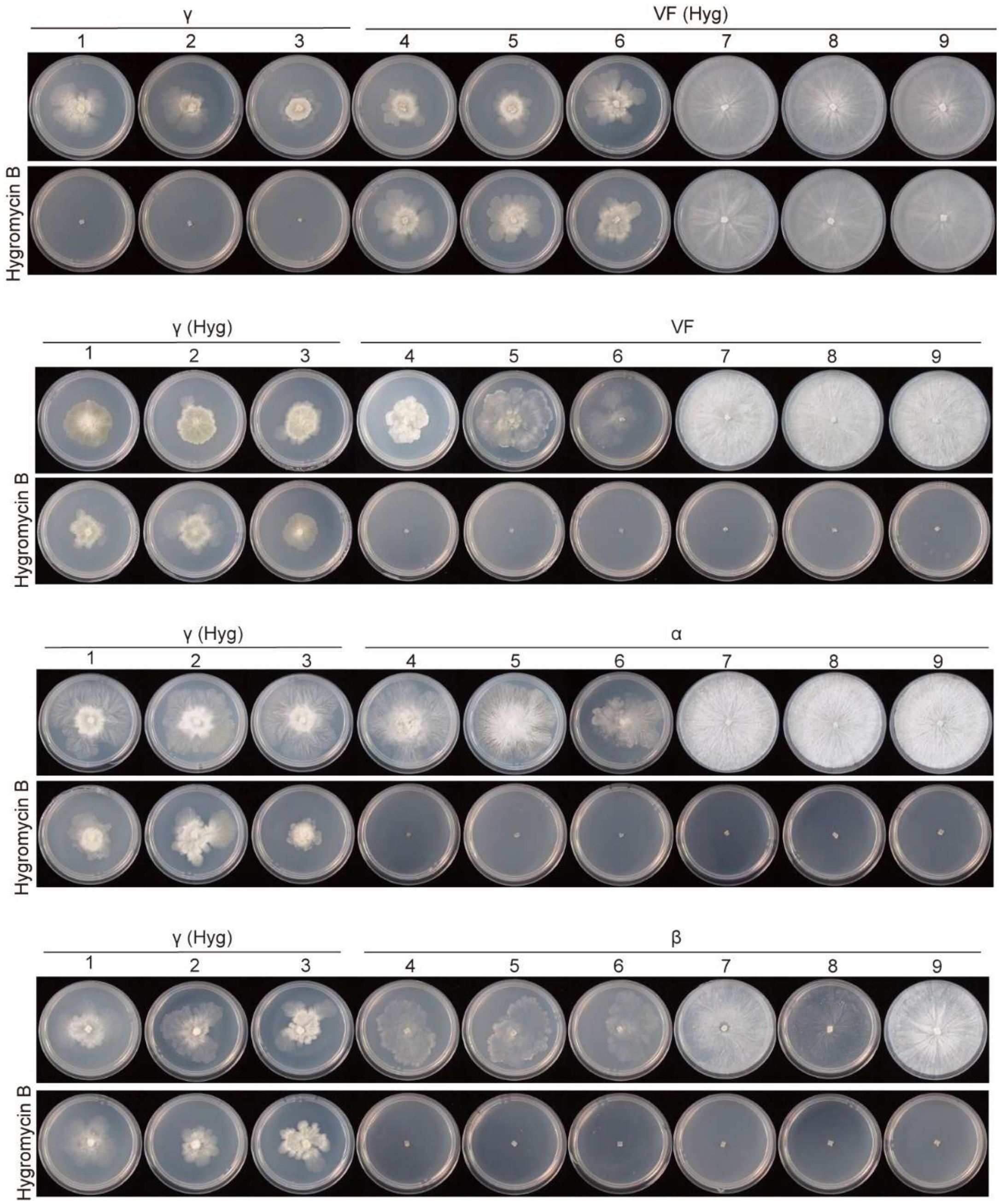

Figure S7. Growth of fungal strains from co-culturing VpHV1-γ variant-infected *V. pyri* strain with isogenic virus-free and α or β variant-infected strains on PDA medium (60 mm plate) with or without hygromycin B. Fungal colonies were photographed at 3 days after co-culturing.

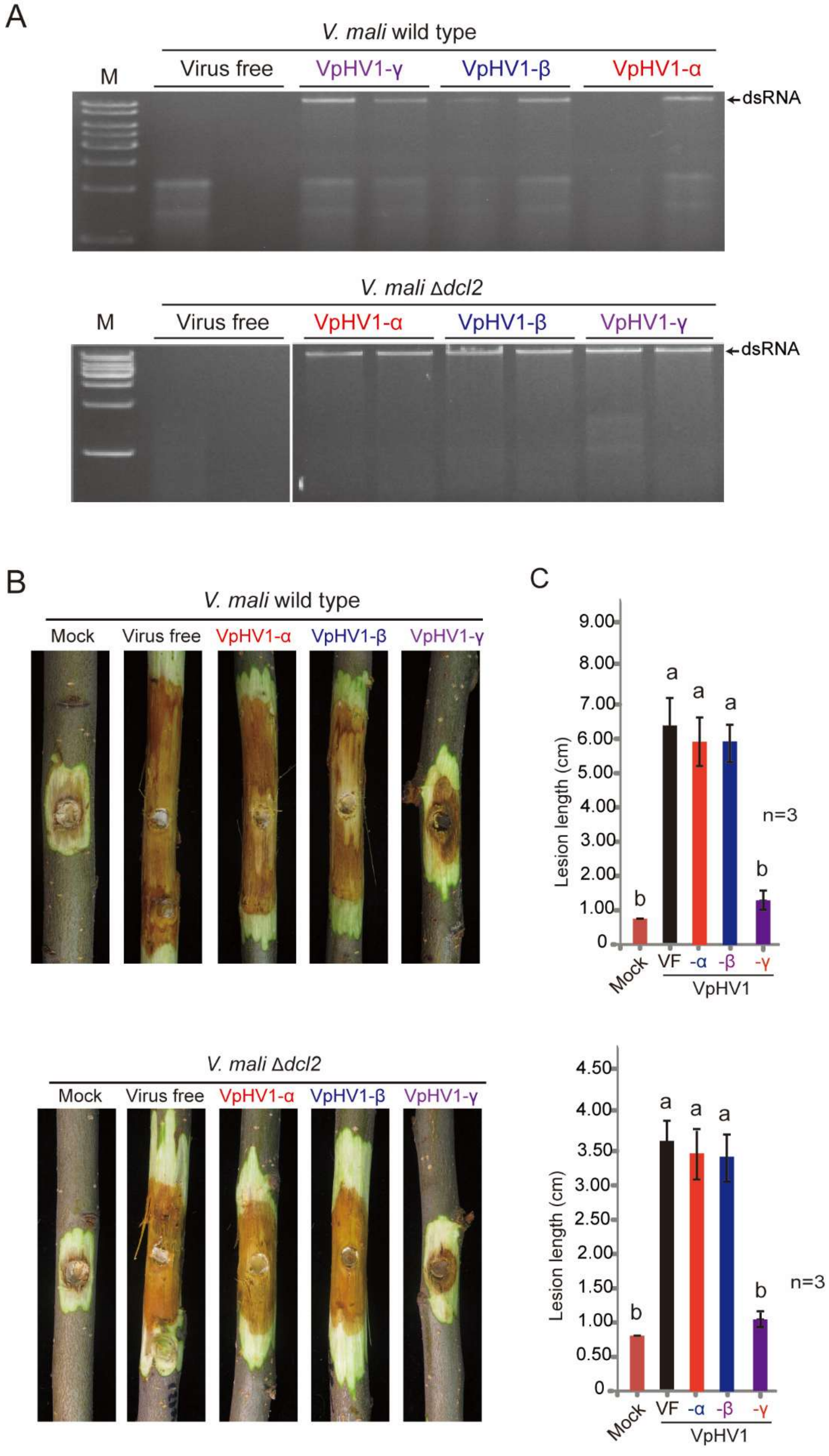
A
V. mali wild type
M
Virus free
VpHV1-γ
VpHV1-β
VpHV1-α
dsRNA
V. mali Δdcl2
M
Virus free
VpHV1-α
VpHV1-β
VpHV1-γ
dsRNA
B
V. mali wild type
Mock
Virus free
VpHV1-α
VpHV1-β
VpHV1-γ
C
Lesion length (cm)
n=3
Mock
VF
-α
-β
-γ
VpHV1
V. mali Δdcl2
Mock
Virus free
VpHV1-α
VpHV1-β
VpHV1-γ
Lesion length (cm)
n=3
Mock
VF
-α
-β
-γ
VpHV1

Figure S8. VpHV1 variant infection and hypovirulence in wild-type and Δ*dcl2* *V. mali* strains. **A.** Viral dsRNA accumulation of VpHV1 variants in *V. mali* strains. **B.** Fungal inoculation assay on apple twigs. Fungal lesions were photographed at 7 days after inoculation. **C.** Lesion length measured on twigs from panel B. Data represent mean ± SD (*n*=3). Different letters indicate significant differences ($P < 0.05$, one-way ANOVA).
