# Supplementary material for: Trade-off between accumulation potential and transmission efficiency in hypovirus variants infecting phytopathogenic fungi
Source: mBio. 2026 Jan 21;17(2):e02922-25. doi: 10.1128/mbio.02922-25 (PMC12892957; doi:10.1128/mbio.02922-25)
Supplement: Supplemental Text — Supplemental methods. [file mbio.02922-25-s0003.pdf]

Material and Methods

Sequence analyses

The identified viral sequences were subjected to alignment and phylogenetic analyses. The N-terminal region of the VpHV1 polyprotein and the RNA-dependent RNA polymerase (RdRP) domain were aligned with other members of the *Hypoviridae* family using MAFFT version 7 (https://mafft.cbrc.jp/alignment/server/), with visualization in Jalview software (v2.11.3.3). A maximum-likelihood phylogenetic tree was constructed using MEGA X [1], with the best-fit substitution model (LG+F+I+G4) selected by ModelFinder in MEGA X.

RT-qPCR, RNA blotting and Western blotting

RT-qPCR were performed using Green qPCR SuperMix (TransGen, China, AQ601-01-V2) on a CFX96™ Real-Time PCR Detection System (Bio-Rad, USA). The 18S gene served as an internal control for normalization. Accession numbers of *V. pyri* AGL1 – 3 are KUI56371.1, KUI59236.1 and KUI56682.1 and DCL1−2 are KUI56844.1 and KUI57727.1.

RNA blot analysis was carried out using Digoxigenin-labeled (DIG, Roche Diagnostics) DNA probes as described in the DIG Application Manual supplied by Roche. Western blot analysis was performed as described previously [2]. The enhanced green fluorescent protein (eGFP) a primary GFP mouse polyclonal antibodies (1:5,000; Proteintech, China) and a secondary HRP-conjugated anti-mouse IgG polyclonal antibody (1:10,000; Abcam, UK). Protein bands were visualized using ChemiDoc Imaging Systems (CLiNX, China).

Pathogenicity assays

Fungal pathogenicity of *V. pyri* and *V. mali* infected with VpHV1 variants was assessed as described previously [3]. The virus-infected and virus-free strains were cultured on PDA medium placed on a benchtop at 24-26°C for 3 days. Detached pear or apple twigs and leaves obtained from Economic Tree Research Field at Northwest A&F University, China, were used to inoculate with mycelial plugs cut from the periphery of the aforementioned fungal colonies. Seven days after inoculation, the length of lesions on pear twigs and the area of lesions on leaves induced by fungal growth were measured. Each inoculation was repeated three times. The surfaces of the twigs and leaves were washed with sterile water and wiped with 75% ethanol before inoculation.

The inoculation materials were placed in a sterile container in a humid environment.

Protein expression in insect cells

Spodoptera frugiperda 9 (*Sf*9) cells (Invitrogen, USA) were cultured in SFX insect medium (HyClone, GE healthcare life science) supplemented with 1% fetal bovine serum (HyClone, USA). To generate recombinant baculovirus, the pQBX plasmid carrying a fusion construct was co-transfected with the linearized Bac563-5T bacmid [4] into *Sf*9 cells using FuGENE HD Transfection Reagent (Promega, USA). At 5 days post-transfection, the supernatant containing recombinant baculovirus was collected by centrifugation at 300 × g for 5 min to remove cell debris. Viral titers were determined by 50% tissue culture infective doses (TCID50) assay [5] after two passages. To examine the protein expression, *Sf*9 cells were seeded in 12-well plates (1×10^6 cells/well), infected with the recombinant baculovirus at an MOI of 3. At 5 days post-infection, the infected cells were lysed using Buffer L (20 mM Tris-HCl, 1 mM EDTA, and 10 mM maltose, pH 7.4) and subjected to Western blot analysis.

Fungal subculture, viral transmission and mycelial compatibility assays

Repeated fungal subcultures of the VpHV1-α-infected strain on PDA medium were performed until phenotypic divergence appeared. Single-spore isolation was conducted to assess vertical transmission. For fungal co-culture assays, two mycelial plugs were placed 1 cm apart on 90 mm PDA plates, as previously described [3]. Programmed cell death (PCD) was assessed 5 days post-co-culturing by staining with 5% Evans blue dye (Fluka, USA) for 10 minutes, followed by three washes with distilled water.

Virus inoculation to plants

Total RNA extracted from virus-infected fungal mycelia was mechanically inoculated to plants by rubbing carborundum-dusted leaves. Concentration of total RNA inoculum for α, β, and γ variants was 5.2, 4.4 and 1.4 μg/μl, respectively.

Observation of fluorescent protein and autophagosomes.

eGFP expression in mycelial cell was observed using a confocal laser scanning microscope (CLSM, Olympus FV3000, Japan) with excitation at 488 nm and emission between 510 to 550 nm. GFP-labelled autophagosomes quantified in 20 cells per replicate, with three biological replicates performed.

Reference

1. Kumar S, Stecher G, Li M *et al.* Mega x: Molecular evolutionary genetics analysis across computing platforms. *Molecular biology and evolution*. 2018;**35**:1547-49
2. Sun L, Suzuki N. Intragenic rearrangements of a mycoreovirus induced by the multifunctional protein p29 encoded by the prototypic hypovirus chv1-ep713. *RNa*. 2008;**14**:2557-71
3. Yang S, Dai R, Salaipeth L *et al.* Infection of two heterologous mycoviruses reduces the virulence of valsa mali, a fungal agent of apple valsa canker disease. *Frontiers in Microbiology*. 2021;**12**:659210
4. Zhang X, Zhao K, Lan L *et al.* Improvement of protein production by engineering a novel antiapoptotic baculovirus vector to suppress the expression of sf-caspase-1 and tn-caspase-1. *Biotechnology and Bioengineering*. 2021;**118**:2977-89
5. Zhang Z, Zhang X, Chen H Tcid50 assay: A simple method to determine baculovirus titer. *Baculovirus: Methods and protocols*, Springer. 267-70
