# Supplementary material for: Trade-off between accumulation potential and transmission efficiency in hypovirus variants infecting phytopathogenic fungi
Source: mBio. 2026 Jan 21;17(2):e02922-25. doi: 10.1128/mbio.02922-25 (PMC12892957; doi:10.1128/mbio.02922-25)
Supplement: Supplemental Tables — Tables S1 to S4. [file mbio.02922-25-s0004.pdf]

**Supplementary Table S1**. List of plant samples and fungal strains used for NGS analysis.

| Sample collection area | [a]Plant species | [b]No. of sample | No. of fungal strains | | No. of *Valsa* strains tested for dsRNA | [d]No. of strains (+) dsRNA |
|---|---|---|---|---|---|---|
| | | | [c]*Valsa* spp. | Others | | |
| Changji City | Apple | 4 | 102 | 3 | 70 | [e]20 |
| | Chinese crabapple | 1 | 24 | 0 | | |
| Aksu City | Xinjiang pear | 17 | 267 | 66 | 144 | 20 |
| | Apple | 3 | 47 | 8 | | |
| | Chinese hawthorn | 1 | 9 | 2 | | |
| Korla City | Xinjiang pear | 17 | 442 | 159 | 311 | 124 |
| | Birchleaf pear | 1 | 21 | 3 | | |
| Total | 5 species | 54 | 912 | 241 | 525 | 164 |

[a]Apple (*Malus domestica*), Chinese crabapple (*M. baccata*), Xinjiang pear (*Pyrus sinkiangesis*), Chinese hawthorn (*Crataegus pinnatifida*) and Birchleaf pear (*P. betulifolia*)

[b]One sample represent a pooled of stems/twigs collected from several tress in an orchard or multiples orchards located in a town.

[c]Changji fungal strains: *Valsa germanica* and *V. malicola*, Aksu and Korla fungal strains: *V. pyri*

[d]dsRNA samples were pooled according to area (three samples: Changji, Aksu and Korla samples) and subjected to NGS analysis.

[e]*V. germanica*

**Supplementary Table S2.** A list of *vic* genes analyzed in this study.

| Gene name | *C. parasitica* Genbank | *Cytospora mali* Genbank | Identity |
|---|---|---|---|
| Vegetative incompatibility protein 1a (*Vic1a-2*) | HG799042 | KUI56542.1<br>KUI55078.1 | 29.38%<br>27.69% |
| Vegetative incompatibility protein 3b (*Vic3b-1*) | tr\|W1IAX8\|W1IAX8_CRYPA | KUI65787.1 | 51.96% |
| partner of vegetative incompatibility protein 6 (*Pix 6-2*) | AET07140.1 | KUI60712.1 | 59.55% |
| Vegetative incompatibility protein 7 (*Vic7a-2*) | r\|A0A6B9UL08\|A0A6B9UL08 | KUI60753.1 | 33.72% |

**Supplementary Table S3.** A list of primers used in this study.

| Clone Name | Primer Name | Oligonucleotide sequence (5’- 3’) |
|---|---|---|
| **Primers used for VpHV1 RT-PCR and PCR** | | |
| | VpHV15'-F | GATCTGACACTTCCCGTGGAGC |
| | VpHV15'-R | GATATGGATCTGACACCGGCACTC |
| | VpHV1M'-F | GAACCTTCAGCACATAAGCAGAGA |
| | VpHV1M'-R | GACGAAACAGAACAAGCAAGTGGT |
| | VpHV13'-F | CGTCATGCTGTCCAGTTGTTTCC |
| | VpHV13'-R | TGCGAATACTGTTTGGCTGTTGG |
| | VpHV1-PolyT | TTTTTTTTTTTTTTTTTTTTTTTCGGAAATTGGTTGACGC CGA |
| | VpHV1-417F | CCCGTGGAGCTAGCAAGGTT |
| | VpHV1-2062R | TCCTATTGTCCCCATCACCAC |
| | VpHV1-673F | TGGTGACACTTCCTTTGCC |
| | VpHV1-930F | GAGGTTCCCAAAGTCCTTG |
| | VpHV1-B-F | GGTGTTTTCGAGCTCTTC |
| **Primers used for VpHV1 full length and RACE** | | |
| | PC3-T7 loop | GGATCCCGGGAATTCGGTAATACGACTCACTATATTTTA TAGTGAGTCGTATTA |
| | PC2 | CCGAATTCCCGGGATCC |
| | VpHV15'RACE-R | CATGTTCTTGGACAAGACCTGATG |
| | VpHV13'RACE-F | GTTGGTACTGGGTAGCAAGAGTGGC |
| | VpHV1-1F | GGGGTTAGAGAACTCTGTGAGTTC |
| | VpHV1-3282R | CCAACAGCACAACAAGTGTTCAC |
| | VpHV1-2725F | GCAGGTCTTTGAGGTGTGTCC |
| | VpHV1M'-R | GACGAAACAGAACAAGCAAGTGGT |
| | VpHV1M'-F | GAACCTTCAGCACATAAGCAGAGA |
| | VpHV1-7553R | TCTGAGCAAGCATCTCCATCTT |
| | VpHV1-6951F | AGGAGGTTGCGACATACACAGG |
| | VpHV1-PolyT | TTTTTTTTTTTTTTTTTTTTTTTCGGAAATTGGTTGACGC CGA |
| **Primers used for RT-qPCR** | | |
| | Dicer1-12F | TAAACTACCCGACTTGAGCG |
| | Dicer1-196R | CCTCTTCATCGTTGCTTCTG |
| | Dicer2-1081F | GGACATCTCTCTCTTTGGTTCTG |

| | | |
|---|---|---|
| | Dicer2-1266R | CGCCTCAAGGACAGCATAAT |
| | Ago1-2985F | CGACAACTCTGACACCACTTC |
| | Ago1-3114R | CTAGATGTACCACATGGTGCTC |
| | Ago2-1038F | GAAACACTCATCACAACGGC |
| | Ago2-1288R | ATAGGTGGGATGGTCTTGGG |
| | Ago3-611F | GAGGTCGCTGGTAAGAAGTC |
| | Ago3-809R | CTCGCTCCTTTGGTTCATC |
| | KUI65787-3b-F | GCACAAGATTACTACCACCAGG |
| | KUI65787-3b-R | ATCGGCACACTCCTCAAGAG |
| | KUI60712-6F | AAATGCGGACAGTTTGGC |
| | KUI60712-6R | GACCTTCTTCTTCTCGTGAGC |
| | KUI60753-7F | CAATCTTGCTGCCATAAGTGG |
| | KUI60753-7R | CAAAGGACGCCAGACAACTC |
| | KUI55078-1F | ATTCTTCTCATCGCCGAGTG |
| | KUI55078-1R | CTGGTGGGCAACTTCTGAAC |
| | KUI56542-1F | GGCTCTATTGTCTTCAAGGTGG |
| | KUI56542-1R | TCGGGAATCAGTCTCTTCATC |
| | 18S-F | AAACTTTCAACAACGGATCTCTTGG |
| | 18S-R | AAATGACGCTCGAACAGGCATG |

**Primers used for transient expression in sf9 cell**

| | | |
|---|---|---|
| pBin61GFP-VpHV1 | pBin61GFP-VpHV1-F | acccccggggtcgacggatccATGTTGAACTATCTTAAAACTCAG |
| | pBin61GFP-VpHV1-R | tctagttcatctagaggatccTCAAGCGTAATCTGGAACATCGTATGGGTAATCAGATGCCATCCAACCGTG |
| pQBX-VpHV1-eGFP | F-BamHI-eGFP-VpHV1-F | ATAAAAAACCGCCACCATGGGATCCATGGTGAGCAAGGGCGAGGAG |
| | R- HindIII -VpHV1-R | TAGCGAATTCCTCGAGAAGCTTTCAAGCGTAATCTGGAACATCGTA |
| pQBX-VpHV1-eGFP-P43 | F-BamHI-eGFP-VpHV1-F | ATAAAAAACCGCCACCATGGGATCCATGGTGAGCAAGGGCGAGGAG |
| | R1- HindIII-eGFP-p43-R | TAGCGAATTCCTCGAGAAGCTTTCATCCTATCCTTGCACTAGGATTTTC |
| pQBX-VpHV1-eGFP-P19 | F-BamHI-eGFP-VpHV1-F | ATAAAAAACCGCCACCATGGGATCCATGGTGAGCAAGGGCGAGGAG |
| | pQBX-P19R | TAGCGAATTCCTCGAGAAGCTTTCAAGCGTAATCTGGAACATCGTATGGGTAAGCCCCCCCAAAGCTTTTAG |
| pQBX-α-mutRVGG | F-BamHI-eGFP-VpHV1-F | ATAAAAAACCGCCACCATGGGATCCATGGTGAGCAAGGGCGAGGAG |
| | L-171-R | GCTCTGATAGGTTCTCGGCTGCCGCCGCAGCCCCCCCAAAGCTTTTAGA |
| | L-171-F | TCTAAAAGCTTTGGGGGGGCTGCGGCGGCAGCCGAGAACCTATCAGAGCTC |
| | R- HindIII -VpHV1-R | TAGCGAATTCCTCGAGAAGCTTTCAAGCGTAATCTGGAACATCGTA |
| pQBX-α-mutRIG | F-BamHI-eGFP-VpHV1-F | ATAAAAAACCGCCACCATGGGATCCATGGTGAGCAAGGGCGAGGAG |

| | | |
|---|---|---|
| pQBX-γ- mutRIG | L-385-R | GATTCCAAATCTATTGCTGCTGCTGCCGCTGCACTAGGATTTTCGGCAAGC |
| | L-385-F | GCTTGCCGAAAATCCTAGTGCAGCGGCAGCAGCAGCAATAGATTTGGAATC |
| | R- HindIII -VpHV1-R | TAGCGAATTCCTCGAGAAGCTTTCAAGCGTAATCTGGAACATCGTA |
| | F-BamHI-eGFP-VpHV1-F | ATAAAAAACCGCCACCATGGGATCCATGGTGAGCAAGGGCGAGGAG |
| | S-171-R | CCATCACCACCGCTGTTGTGGCTGCCGCCGCAGCCCCCCCAAAGCTTTTA |
| | S-171-F | TAAAAGCTTTGGGGGGGCTGCGGCGGCAGCCACAACAGCGGTGGTGATGG |
| | R- HindIII -VpHV1-R | TAGCGAATTCCTCGAGAAGCTTTCAAGCGTAATCTGGAACATCGTA |
| **Primers used for infectious clone** | | |
| | pCPXHV1-NotI-T7F | TACTACTTTTAGGTACGCGGCCGCTAATACGACTCACTATAGGGGGGGTTAGAGAACTCTGTGAGTTC |
| | pCPXHV1-HindIII-T7R | GACGTGGGAGATCAGGTCAAGCTTGTTAACACTAGTTTTTTTTTTTTTTTTTTTTTTTTCGGAAATTG |
| | VpHV1-1F | GGGGTTAGAGAACTCTGTGAGTTC |
| | VpHV1M'-R | GACGAAACAGAACAAGCAAGTGGT |
| | VpHV1M'-F | GAACCTTCAGCACATAAGCAGAGA |
| | VpHV1-PolyT | TTTTTTTTTTTTTTTTTTTTTTTCGGAAATTGGTTGACGCCGA |
| **Primers used for autophage detection** | | |
| | pCPX-ATG8-eGFP-F | TTTTAGGTACGCGGCCGCACCATGCGATCCAAATTCAAG |
| | pCPX-ATG8-eGFP-R | CTCACCATGGTGTTAACAAGCTTAGTCGACTCGAAGCCTCCA |
| **Primers used for Northern blot probes** | | |
| Probe1 | VpHV1-931F | GAGGTTCCCAAAGTCCTTGG |
| Probe1 | VpHV1-1448R | CAGGGTGATCTACCAGATGGTAG |
| Probe2 | VpHV1M'-F | GAACCTTCAGCACATAAGCAGAGA |
| Probe2 | VpHV1M'-R | GACGAAACAGAACAAGCAAGTGGT |
| **Primers used for species identification** | | |
| | ITS1 | TCCTCCGCTTATTGATATGC |
| | ITS4 | GGAAGTAAAAGTCGTAACAAGG |

**Supplementary Table S4.** A list of plasmid constructs generated in this study.

| Construct | Insertion (region)[a] | Plasmid vector[b] | Restriction sites |
|---|---|---|---|
| GFP-VpHV1-α | VpHV1-α(377-2197nt) | pBin61-GFP | *BamH*I |
| GFP-VpHV1-β | VpHV1-β(377-1852nt) | pBin61-GFP | *BamH*I |
| GFP-VpHV1-γ | VpHV1-γ(377-1063nt) | pBin61-GFP | *BamH*I |
| pQBX-VpHV1-α | eGFP-VpHV1-α (377-2197nt) | pQBX | *BamH*I and *Hind*III |
| pQBX-VpHV1-β | eGFP-VpHV1-β (377-1852nt) | pQBX | *BamH*I and *Hind*III |
| pQBX-VpHV1-γ | eGFP-VpHV1-γ (377-1063nt) | pQBX | *BamH*I and *Hind*III |
| pQBX-VpHV1-α-p19/24 | eGFP-VpHV1-α-p19/24 (377-1039nt) | pQBX | *BamH*I and *Hind*III |
| pQBX-α-p43/24 | eGFP-VpHV1-α-p43/24 (377-2197nt) | pQBX | *BamH*I and *Hind*III |
| pQBX-α-p19/48 | eGFP-VpHV1-α-p19/48 (377-2197nt) | pQBX | *BamH*I and *Hind*III |
| pQBX-α-p67 | eGFP-VpHV1-α-p67 (377-2197nt) | pQBX | *BamH*I and *Hind*III |
| pQBX-α-p43 | eGFP-VpHV1-α-p43 (377-1039nt) | pQBX | *BamH*I and *Hind*III |
| pQBX-β-p54 | eGFP-VpHV1-β-p54 (377-1852nt) | pQBX | *BamH*I and *Hind*III |
| pQBX-γ-p25 | eGFP-VpHV1-γ-p25 (377-1063nt) | pQBX | *BamH*I and *Hind*III |
| pCPX -ATG8-eGFP | ATG8 coding region | pCPX-HY2 | *Not*I and *Hind*III |
| pCPX-VpHV1 | VpHV1 full length | pCPX-HY2 | *Not*I and *Hind*III |
